# Conditional Variational AutoEncoder to Predict Suitable Conditions for Hydrogenation Reactions

**DOI:** 10.3390/molecules31010075

**Published:** 2025-12-24

**Authors:** Daniyar Mazitov, Timur Gimadiev, Assima Poyezzhayeva, Valentina Afonina, Timur Madzhidov

**Affiliations:** 1 A.M. Butlerov Institute of Chemistry, Kazan Federal University, Kremlevskaya Str. 18, 420008 Kazan, Russia; daniyarttt@gmail.com (D.M.); valiaafo@yandex.ru (V.A.); 2 Federal Research Center “Kazan Scientific Center of the Russian Academy of Sciences”, Lobachevskogo Str. 2/31, 420111 Kazan, Russia; 3 Chemistry Solutions, Elsevier, London EC2Y 5AS, UK; tmadzhidov@gmail.com

**Keywords:** conditional variational autoencoder, hydrogenation reactions, hydrogenolysis, H_2_-mediated reactions, condensed graph of reaction, reaction condition prediction

## Abstract

Reaction conditions (RCs) are a crucial part of reaction definition, and their accurate prediction is an important component of chemical synthesis planning. The existence of multiple combinations of RCs capable of achieving the desired result complicates the task of condition recommendation. Herein, we propose a conditional variational autoencoder (CVAE) generative model to predict suitable RCs. The CVAE model has been customized to generate diverse sets of valid conditions, ensuring high flexibility and accuracy, while circumventing the necessity for enumeration or combinatorial search of potential RCs. The efficacy of the CVAE approaches was evaluated using hydrogenation reactions and other H_2_-mediated reactions, predicting the set of catalysts, additives (acid, base, and catalytic poison), ranges of temperature, and pressure. The CVAE models predicted conditions with different “heads”, each corresponding to specific condition components, and their respective losses. CVAE models were tested on two datasets: a small one containing 31K reactions with 2232 potential conditions’ combinations and a big one having 196K reactions with ~7 × 10^42^ potential conditions’ combinations to evaluate the model’s ability to predict varying complexity and diversity conditions. To optimize the accuracy of the models, we experimented with three latent distribution variants—Gaussian (g-CVAE), Riemannian Normalizing Flow (rnf-CVAE), and Hyperspherical Uniform (h-CVAE). In our experiments, the h-CVAE model demonstrated robust overall performance, making it the optimal choice for scenarios requiring high accuracy across multiple top-k predictions. Benchmarking analyses demonstrated the high performance of the CVAE models compared to state-of-the-art reaction condition prediction approaches.

## 1. Introduction

Synthetic organic chemistry’s main task is to synthesize the desired chemical compound. In recent years, automated computer-aided synthesis planning (CASP) has emerged as a promising approach for the design of synthetic routes for new chemicals [1,2,3]. This advancement has been significantly supported by extensive research in retrosynthesis prediction [4,5] and the development of machine learning algorithms designed to identify the most appropriate synthetic pathways. Selecting suitable reaction conditions (referred to as RC), including catalysts, reagents, and solvents, as well as other operating conditions, such as temperature and pressure, is crucial for synthetic chemists. It might be important for maximizing the synthetic route’s overall yield and selectivity, minimizing purification costs, chemical consumption, and environmental impact of the synthesis. Even minor changes in RC can determine the success or failure of a reaction.

Complicated interactions between the chemicals involved make RC prediction so difficult. For example, the compatibility between the reactants and the solvent is essential for a successful reaction; they should not react and generate undesired byproducts. On the other hand, the development of solutions for RC prediction is limited by the availability of high-quality RC datasets. In large commercial reaction databases, the data on conditions is sparse and lacks negative examples. On the contrary, the datasets obtained by high-throughput experimentation (HTE) lack diversity. Thus, most publications on condition prediction [6,7,8,9,10,11] obtained data from commercial databases like Reaxys^®^ (Copyright © 2021 Elsevier Life Sciences IP. Reaxys^®^ is a trademark of Elsevier Life Sciences IP Limited). Recently, Wang et al. [12] and Wigh et al. [13] introduced open-source standardized benchmark datasets for condition prediction (USPTO-Condition and ORDerly-condition, respectively) based on the open USPTO reaction data [14]. However, testing the RC prediction model is also challenging as multiple RC combinations can achieve the desired output. There is no clear one-to-one or many-to-one correspondence between the reaction equation and suitable conditions. Moreover, if the model predicts some conditions that do not match the ones used in the ground truth dataset, this does not mean that the prediction is wrong. It may well be that such conditions achieve the desired outcome, but they were not tested for the given reaction.

Over the past few decades, numerous approaches for predicting suitable RC have been developed. Initially, studies were mainly focused on predicting specific agents (solvent, catalyst, or reagent) for reactions within a single reaction class [15,16,17,18,19]. For example, Struebing et al. [15] used a combination of quantum chemical modeling and linear regression to identify the most suitable solvents for Menshutkin reactions. Marcou et al. [16] used different machine learning (ML) models and descriptors to predict solvent and catalyst classes for Michael addition reactions. Lin A. et al. [17] used the similarity-based approach to predict general catalyst classes for deprotection reactions under catalytic hydrogenation conditions. Segler and Waller [18] used a knowledge graph built on a big reaction database and combined it with chemical reasoning to identify potential reagents and catalyst combinations for wide reaction classes.

Unlike the models mentioned above, which predict only one selected component of condition components, Gao et al. [6] reported a deep neural network (referred to as “Model from ASKCOS”) for RC prediction, agnostic of reaction class, that predicted the catalysts, reagents, solvents, and temperature. The performance of the Gao et al. [6] model was quite reasonable: for the test set reactions, experimentally used catalysts, reagents, and solvents were found in 57% of the top-10 predictions. However, the authors also admitted that the model quality was similar to the nearest neighbor model.

Maser et al. [11] studied the roles of different species in reactions (such as metals, ligands, bases, solvents, and additives) and developed multi-label classification models based on graph networks with attention mechanisms for predicting reaction conditions. The model had reasonable quality when it faced a training set with a small sample size and a limited number of molecular graphs involved in the reaction samples. However, it was unsuitable for modeling using more complex, large-scale, and diverse reaction condition datasets.

Afonina et al. [10] developed the Likelihood Ranking Model (referred to as LRM), delivering a list of conditions ranked according to the model confidence. The publication aimed to predict the reaction catalyst, general reagent type (acid, base, catalytic poison), as well as temperature and pressure represented in binned form (low, medium, high). The authors trained a simple shallow neural network on the obtained multi-hot condition vector to predict probabilities of certain bits corresponding to condition components. Then, possible conditions were ranked using likelihood functions obtained from the probabilities. Despite good performance achieved for hydrogenation reactions, combinatorial enumeration of all possible conditions is impractical for big databases with diverse conditions.

Kwon et al. [9] developed a generative conditional variational autoencoder (CVAE) augmented with a graph neural network (Message Passing Neural Network) predicting multiple RC through repeated sampling from a Gaussian prior distribution. Despite the approach being quite reasonable and having many positive implications, it was only tested on relatively small datasets of cross-coupling reactions.

The generative approach based on CVAE [20,21] has significant benefits over all other approaches described above: it does not use any possible conditions enumeration to assess certain conditions’ applicability. Instead, it directly samples RC using knowledge learned from existing reaction datasets. Importantly, only the CVAE models explicitly utilize the experimentally recorded reaction conditions as an input during training: the encoder receives both the reaction representation and the corresponding reaction conditions vector, enabling it to learn a latent distribution over feasible reaction conditions. During inference, however, the decoder takes only the representation of the chemical transformation, while the condition vector is replaced with a random sample drawn from the prior distribution. Accordingly, the CVAE is designed to generate combinations of RC naturally by sampling from the prior distribution and, therefore, can suggest multiple possible sets of reaction conditions for a given reaction, accounting for the fact that there might be more than one suitable way to carry out a chemical transformation. For a query reaction, multiple predictions can be obtained by repeated sampling from the distribution. Thus, its speed is limited only by the number of conditions to be returned. Also, such an approach does not have limitations on reaction space size or the potential number of conditions that can be used.

The primary goal of this work is to overcome deficiencies of the previously developed condition ranking model (LRM [10]), which was caused by the impossibility to rank large potential reaction condition spaces. As a remedy, we explored RC generation by CVAE models. The developed CVAE models were evaluated on hydrogenation reactions (here, under hydrogenation, we mean any reaction in the presence of H_2_), which are commonly employed in chemical synthesis and are particularly significant in medicinal chemistry. We tested CVAE models on two datasets: the first one is small (referred to as dataset S), containing 31K reactions with 2232 potential conditions’ combinations (notice that not all conditions were actually used in the dataset, see Section 3.1.3). The proposed CVAE models trained on dataset S were primarily developed for comparative analysis with our previous LRM [10], enabling an evaluation of the performance differences between traditional and generative approaches. The second dataset is a big one (dataset B), having 196K reactions with ~7 × 10^42^ potential conditions’ combinations to evaluate the model’s ability to predict conditions of varying complexity and diversity. The predicted outputs of the models were the set of catalysts, additives (acid, base, catalytic poison), ranges of temperature, and pressure, since the success of these reactions depends heavily on these factors. Enumeration of all possible combinations of conditions gives rise to the above-mentioned numbers of potential conditions. It is essential to note that solvent and other additive predictions are not performed in this study, as in the article [10].

**Figure 1:**
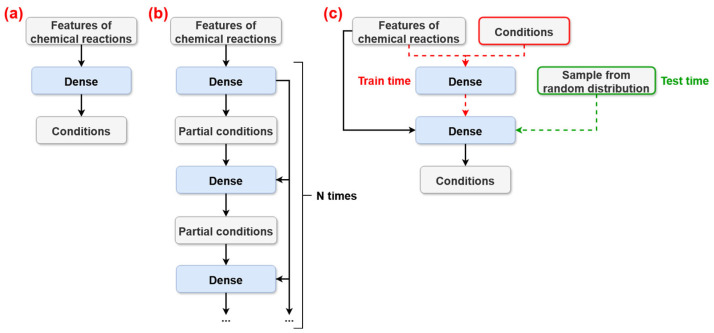
Schematic illustration of the high-level architectures of the following models compared in the article: (**a**)—LRM proposed by Afonina V.A. et al. [10]; (**b**)—Model from ASKCOS proposed by Gao et al. [6]; and (**c**)—our CVAE-based model. “Dense” represents one or more dense layers of neural networks. “Partial conditions” is the output layer containing only certain condition components (e.g., only catalyst or temperature). Only the CVAE architecture incorporates the RCs as an input during training (red dashed lines), enabling learning of a latent distribution over feasible reaction conditions. During inference, these inputs are replaced by random samples drawn from a prior random distribution (green dashed lines), allowing the model to generate multiple possible condition combinations.

While our proposed approach builds on the generative paradigm previously explored by Kwon et al. [9], we introduced several methodological advances to ensure component-specific handling, latent space modeling, and scalability with respect to dataset size. In contrast to Kwon et al. [9], our CVAE models predicted conditions with different “heads”, each corresponding to specific condition components, and their respective losses. This model architecture facilitates targeted learning for each component’s unique characteristics, improves internal consistency among generated outputs, and decreases the chance of incompatible condition combinations. Moreover, we thoroughly compared several latent space formulations, including the standard Gaussian distribution (g-CVAE) and two advanced alternatives—Riemannian Normalizing Flow (rnf-CVAE) and hyperspherical uniform (h-CVAE), which offer improved flexibility for modeling complex and non-linear condition distributions. The proposed three CVAE models (g-CVAE, rnf-CVAE, and h-CVAE) were compared against state-of-the-art reaction condition prediction approaches: k Nearest Neighbors [10], Gao et al.’s [6], and Afonina V.A. et al.’s [10] models, to evaluate their relative performance (schematic illustration of the architectures of the models is presented in Figure 1).

## 2. Results and Discussion

### 2.1. Performance of the Models Trained on Dataset S

The predictive performance of the CVAE models was first evaluated using the small dataset S described in [10], which contains 31,381 single-step reactions performed in the presence of H_2_ with fully defined conditions. The test set for dataset S was constructed by randomly selecting chemical transformations with only one reported set of conditions, resulting in 3692 reactions and 2232 possible combinations of RCs. More detailed information about dataset S is provided in Section 3. The CVAE models were designed to predict key condition components simultaneously, including catalyst, ranges of temperature and pressure, and additives (acid, base, or catalytic poison, with other additives and solvents ignored). Each additive type was encoded as an independent binary indicator, such that the model predicts only its presence or absence rather than a specific compound.

The impact of different latent space distributions on model accuracy was determined by assessing three CVAE variants—g-CVAE, rnf-CVAE, and h-CVAE. The performance of these generative models was benchmarked against other models: the Null model, the k Nearest Neighbors model (referred to as kNN), the Likelihood Ranking Model (LRM) from Afonina V.A. et al. [10], and the model from ASKCOS proposed by Gao et al. [6]. The performance of the models was assessed using the “precision at k” (p@k) metric, which quantifies the percentage of test reactions for which at least one experimentally recorded combination of RCs appears within the top-k generated predictions (see Section 3.5).

**Figure 2:**
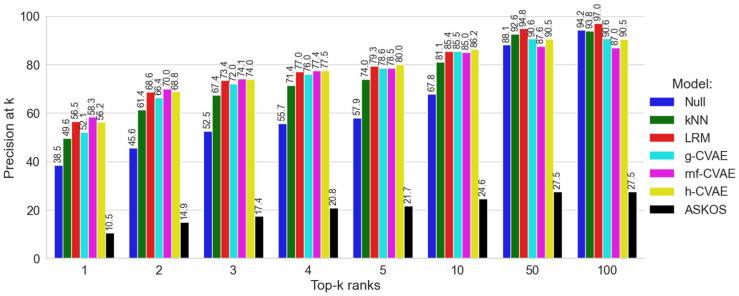
Comparisons of all models on dataset S.

The Null model, which predicts reaction conditions based on their frequency in the training dataset S, achieved a precision of 38.5% on the test set according to the point estimate (Figure 2). The kNN model (Figure 2) achieved a p@1 value of 49.6%, overcoming the Null model. However, as k increases (i.e., as more top-ranked predictions (top-k) are considered), the p@k values for the kNN model consistently remained lower than those of the CVAE model and LRM. The model from ASKCOS demonstrated the lowest p@k values among the compared approaches. This observation can be attributed to the diversity of reaction types in the ASKCOS model’s training set [6], many of which did not involve catalysts—a key determinant in catalytic hydrogenation reactions studied here. Based on the point estimates from the single test set, the rnf-CVAE model, h-CVAE model, and LRM exhibited the highest p@k values, with the correct experimentally observed combination of RC components appearing as the top-1 prediction in 56–58% of test cases (Figure 2). The g-CVAE model achieved a lower p@1 value of 52.1% compared to the other CVAE variants, suggesting that a simple Gaussian distribution in the latent space may be less suitable for modeling the diversity of reaction conditions in this dataset. Thus, based on the results obtained for dataset S, it was established that our previously proposed Likelihood Ranking Model [10] and the CVAE models with non-Gaussian prior distributions demonstrated higher p@k values compared to other models (Null, g-CVAE, kNN [10], and the model from ASKCOS proposed by Gao et al. [6]).

In the subsequent analysis, the predictive accuracy of individual condition components was examined in detail, including catalyst, additives (acid, base, and poison), range of temperature, and pressure. Table 1 presents the precision results for predicting catalyst (c@k), additives (ad@k), range of temperature (t@k), and pressure (p@k) using three CVAE models on dataset S, evaluated at the top-1, top-3, and top-10 ranks. If a reaction condition included multiple additives (catalytic poison, acid, or base), ad@k was evaluated in the same way as p@k, meaning that only a complete match of compounds was considered correct. The dataset S does not contain RCs where both an acid and a base are present simultaneously (see Section 3.1.2); each RC always included a single catalyst, as reactions involving more than one catalyst were excluded. When evaluating predictions for a specific category within a combination of conditions, only the corresponding part of the binary string (see Section 3.3.2) associated with that category was compared. Solvent prediction was not addressed in this study. The results of this analysis are summarized in Table 1. Based on the point estimates from the single test set, all three models (g-CVAE, rnf-CVAE, and h-CVAE) achieved relatively high values of metrics across all condition components, with minimal differences between them. For example, the rnf-CVAE model achieved the highest c@1 value (80.17% and 85.37%) at lower ranks, while h-CVAE and g-CVAE models yielded slightly lower values (79.39% and 79.04%, respectively). A notable observation is that all three CVAE models showed high precision for recovering the correct catalyst, additive components, range of temperature, and pressure at the top-10 level, with point estimates exceeding 93%.

### 2.2. Performance of the Models Trained on Dataset B

The performance of the CVAE models was further evaluated using dataset B, a significantly more extensive and diverse dataset comprising 157,051 reactions in the training set and 39,261 H_2_-mediated reactions in the test set. The list of catalysts was considerably broader, and acid, base, and catalytic poison were represented as individual compounds rather than generalized classes, as was performed in dataset S. In contrast to dataset S, which included reactions with fully specified condition components, dataset B contained explicit temperature and pressure values only for about 39% of the data (see Section 3.3.3). For evaluation of models, the test set for dataset B was constructed exclusively from reactions for which temperature and pressure were explicitly recorded, ensuring that the p@k metric reflected comparison against complete and unambiguous condition sets.

**Figure 3:**
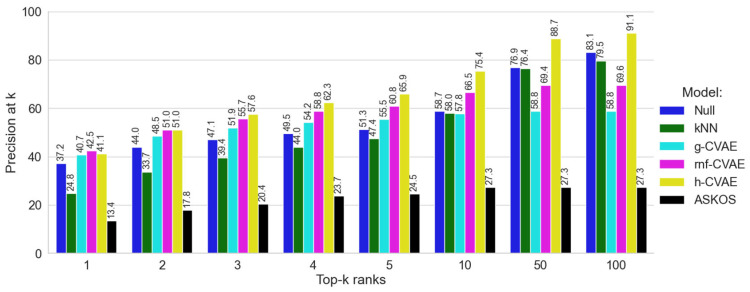
Comparison of all models on dataset B.

The condition space for dataset B is vast, with approximately 7 × 10^42^ possible condition combinations. Given the vast space, combinatorial enumeration of all possible RCs, as required by the LRM, is impractical for large databases with diverse conditions. Therefore, despite the competitive point estimates achieved by the LRM on the dataset S, the LRM could not be applied to dataset B. Consequently, the performance of the CVAE models was benchmarked against the Null model, kNN, and the model from ASKCOS [6]. The ensuing results are presented in Figure 3.

The Null model, which predicts conditions based on their frequency in the training dataset B, achieved precision at top-1 rank (37%) (Figure 3). It is worth noting that training dataset B includes some records with unknown temperature or pressure values (see Supplementary Materials, Figures S1 and S2). Such a high value of the metric for the Null model is due to the fact that incomplete records were excluded when constructing this model (see Supplementary Materials, Figures S1 and S2). As demonstrated in Figure 3, the model from ASKCOS exhibited limited performance, with precision ranging between 13% and 27% across different k values. The kNN model demonstrated a more consistent improvement across ranks, starting around 24.8% at top-1 and gradually improving to 79.5% at top-100, reflecting its ability to recover correct conditions from the training set (Figure 3). For such a diverse dataset, it becomes quite challenging for models to beat the popularity-based Null model, and ranking kNN and ASKOS models fail here.

Initially, at the top-1 and top-2 ranks, h-CVAE, rnf-CVAE, and g-CVAE demonstrated comparable predictive abilities, slightly above those of the Null model, with p@k values of approximately 41% (for top-1) and 51% (for top-2), as illustrated in Figure 3. However, as the rank increases, a notable difference in performance becomes evident, starting from top-4, particularly for the h-CVAE model. The h-CVAE model consistently showed higher point estimates for precision across all k values. Specifically, the h-CVAE attained a precision of 75.4% at top-10 and over 88% at top-50 and top-100 ranks. In contrast, the g-CVAE and rnf-CVAE models exhibited a different pattern, attaining 58.8% and 69.4% precision by top-50, respectively. Their performance remained at these levels without significant further enhancement.

Thus, benchmarking analyses demonstrated that our CVAE models achieved higher precision values compared to state-of-the-art reaction condition prediction approaches. The results indicate that while the g-CVAE showed performance superior to the Null model, advanced latent space modeling techniques can be associated with improvements in reaction condition prediction. Specifically, the rnf-CVAE achieved the highest p@1 value; however, its performance appeared less competitive for predictions beyond the first rank. In contrast, the h-CVAE model demonstrated robust overall performance, making it the optimal choice for scenarios requiring high accuracy across multiple top-k predictions.

Subsequently, an evaluation was conducted to ascertain the efficacy of the models in predicting condition components. Table 2 presents the precision results for predicting catalyst (c@k), additives (ad@k), range of temperature (t@k), and pressure (p@k) using three CVAE models on dataset B, evaluated at the top-1, top-3, and top-10 ranks. Similar to the results observed with dataset S, based on point estimates, all three models (g-CVAE, rnf-CVAE, and h-CVAE) achieved relatively high precision across all condition components at top-1, with only minor differences between them. The rnf-CVAE model consistently achieved higher point estimates than g-CVAE and h-CVAE for predicting catalysts (c@3 = 83.41%) and additives (ad@3 = 84.82%) at top-3 (see Table 2). The h-CVAE model demonstrated strong performance for predicting the range of temperature and pressure conditions at top-3. By top-10, this model achieved high precision in recovering all condition components, whereas the g-CVAE exhibited a somewhat lower performance.

**Figure 4:**
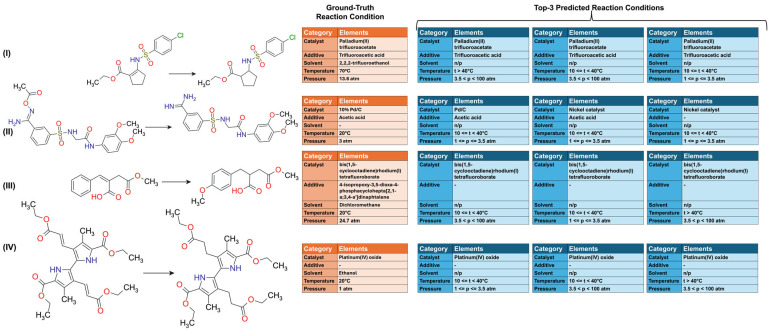
Prediction of reaction conditions for H_2_-mediated reactions. Example of h-CVAE model predictions (top-3) for test set examples compared with recorded conditions. Solvent information is shown for reference only and was not a predicted variable (orange: recorded conditions; blue: predicted conditions). Dataset B. n/p—not predicted.

A qualitative analysis was conducted by comparing predicted and experimentally recorded reaction conditions for H_2_-mediated reactions from the test set. Although all three CVAE models exhibited comparable performance at top-1, the h-CVAE model was selected for this analysis due to its high performance at different top-k ranks, where it consistently demonstrated the highest precision in predicting the most likely condition combination. Utilizing the h-CVAE model trained on dataset B, the top-3 predicted reaction conditions for four hydrogenation reactions from the test set were identified (see Figure 4, blue tables). The experimentally recorded conditions of RCs in Figure 4 (orange table) contained more additives than those given in the predicted conditions because the primary focus of the model was on predicting catalysts, acids, bases, and catalyst poisons. For example, in experimentally recorded conditions of reaction I, the solvent specified was trifluoroacetic acid; however, the prediction of solvents and other additives was not performed in this study. As demonstrated by the examples (Figure 4), the model successfully generated a combination of RCs for each reaction. The h-CVAE model’s predicted ranges for temperature and pressure generally encompassed the actual experimental value. For pressure, this indicates correct order-of-magnitude classification (Figure 4). The CVAE models trained on dataset B enabled the model to predict specific compounds that fulfilled distinct roles, such as acids, bases, or catalyst poisons, depending on the reaction context. The h-CVAE model demonstrated a reasonably good alignment with the ground truth, consistently predicting the correct catalysts and additives for each reaction. The model demonstrated an ability to accurately identify the requisite additive in several instances, even when such conditions were underrepresented in the training set. In reaction IV (see Figure 4), the predicted conditions closely matched common reaction conditions frequently observed in the training set (according to Supplementary Materials, Figures S1 and S2). However, the model also demonstrated a good degree of success in its prediction of rare conditions for reactions I and III (Figure 4). The additional examples of evaluated reactions and their corresponding predictions are presented in Supplementary Materials, Table S1.

**Figure 5:**
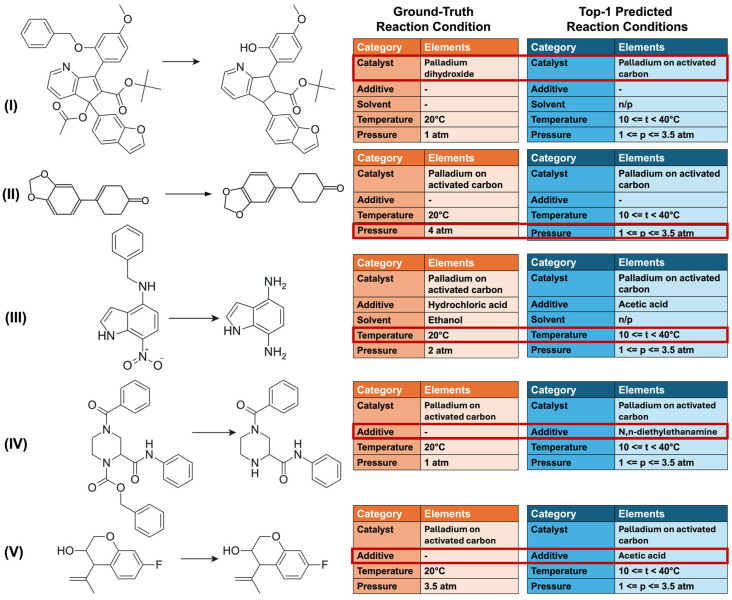
Condition prediction for catalytic reactions under H_2_ (cases with near-complete match). Example of h-CVAE model predictions (top-1) for test set examples where the model correctly identified the combination of reaction conditions except for one component, compared with recorded conditions (orange: recorded conditions; blue: predicted conditions). Dataset B.

In conclusion, we performed a qualitative analysis of top-1 predictions in cases where the h-CVAE model correctly identified the combination of RCs, except for one condition component. Specifically, the model accurately predicted temperature and pressure ranges but proposed a different catalyst (see Figure 5, reaction I). A more detailed analysis of such cases (see Supplementary Materials, Table S2) demonstrated that the proposed catalyst typically belonged to the same active transition metal as the ground truth catalyst, while differing in its support, ligand environment, or oxidation state. This analysis illustrates that the model tends to remain within a chemically related region of the condition space. For example, in reaction I (Figure 5), the h-CVAE recommended palladium on activated carbon at top-1, whereas the ground truth conditions specified palladium dihydroxide; notably, the latter was ranked as top-2 by the model (see Supplementary Materials, Table S2). Likewise, deviations in the predicted temperature or pressure usually corresponded to neighboring ranges rather than entirely different ranges (see Figure 5, reactions II and III). In some cases, the h-CVAE correctly predicted all reaction conditions but additionally suggested the presence of an acid or base, although the reported experimental RCs did not require such additives (see Figure 5, reactions IV and V). It is not possible to objectively determine the practical feasibility or optimality of non-matching predicted conditions without experimental validation. Therefore, the analysis of such predictions is limited to a qualitative assessment of model behavior in the condition space. Experimental verification remains a necessary step beyond the scope of this work.

## 3. Materials and Methods

### 3.1. Dataset Acquisition and Curation

#### 3.1.1. Chemical Structure Curation

Reaxys^®^ (Copyright © 2021 Elsevier Life Sciences IP. Reaxys^®^ is a trademark of Elsevier Life Sciences IP Limited, Elsevier, London, UK) was the primary source of reaction content in this publication. Two datasets of hydrogenation reactions, differing in size and complexity, were used for modeling. In this work, by hydrogenation we mean any reaction occurring in the presence of H_2_; therefore, the datasets included both the addition reactions of H_2_ to unsaturated bonds and other H_2_-dependent reactions, such as hydrogenolysis and the reduction in heteroatom groups (e.g., nitro groups). For both datasets, a similar data curation procedure was applied. Compound structures were standardized according to the protocol described in the article [22]. The detailed steps for chemical structure curation can be found in Appendix A.1. We used the CGRtools library (version 3.1) [23] for functional group normalization, aromatization, removing explicitly specified hydrogen atoms, and duplicate cleaning. Atom-to-atom mapping of reactions was performed using a ChemAxon Standardizer (JChem 19.4.0, 2019) [24].

#### 3.1.2. Reaction Condition Curation

The curation of reaction conditions was conducted by the protocol described in the article [10]. The detailed steps for reaction condition curation can be found in Appendix A.2. Temperature and pressure data were extracted from the numerical fields, and if there was no information, data from text fields were extracted. These values were then binarized into ranges (see Section 3.3.2 and Section 3.3.3).

After standardizing the names of compounds, their roles in the hydrogenation reaction were redefined and reassigned as solvents, additives (acid, base, or catalytic poisons), and catalysts. This process was semi-automated, as it was initially based on the frequency of occurrence in the relevant database fields. For example, if a compound appeared most frequently in the catalyst field, it was designated as a catalyst. Thus, each compound in the dataset was assigned only one role. Additionally, catalysts were further annotated based on the presence of metals commonly involved in catalysis. Another key standardization step was the conversion of sets of individual compounds corresponding to complex composite catalysts to a standard name. A notable example is the Lindlar catalyst.

The roles of acid and base were assigned based on known or predicted pKa values for acids and conjugate pKa values for bases. Compounds containing elements that usually reduce the catalytic activity of hydrogenation reactions were classified as catalytic poisons.

Reactions were excluded if RCs (1) involved more than one catalyst, or (2) recorded both an acid and a base simultaneously under the conditions. Subsequently, a second round of duplicate removal was conducted.

#### 3.1.3. Datasets

The first dataset (dataset S) was formed based on the data used in the article [17]. Initially, the dataset of hydrogenation reactions was extracted from the Reaxys^®^ database (downloaded in 2013) through a formal criterion of a “one-step reaction with H_2_ in reagents/catalyst fields”. To avoid ambiguity, the terminology used in this manuscript is defined as follows: a “transformation” refers to a set of reactants and products, while a “reaction” denotes a transformation together with the specific reaction conditions used. Dataset S comprised 142,111 chemical transformations proceeding under ca. 271,000 recorded RCs. Finally, reactions were selected based on the condition information that was decided to include in the output (described in Section 3.3.2). After data curation and dataset preparation, dataset S contained 29,609 chemical transformations (31,381 reactions) with fully defined conditions.

**Figure A1:**
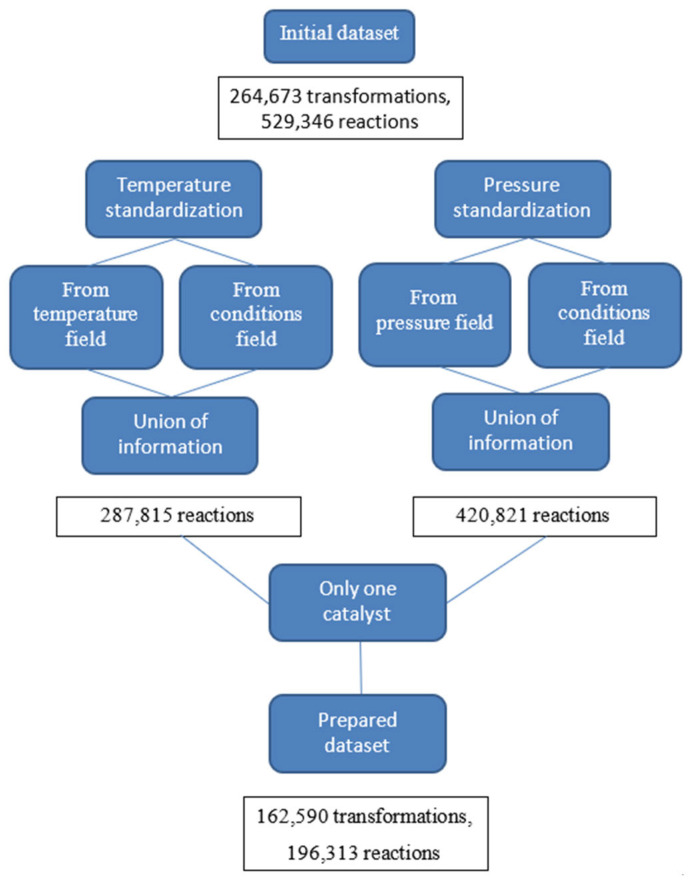
Scheme of conditions standardization.

The second dataset (dataset B) was compiled by extracting 264,673 single-step catalytic hydrogenation and 402,000 RC records from the Reaxys^®^ database (downloaded in 2017) via the Reaxys^®^ API. Following the curation and selection of reactions with information about desired conditions (described in Section 3.3.3), dataset B included 162,590 chemical transformations (196,313 reactions, see Appendix A.2, Figure A1). Unlike dataset S, which contained reactions including all required condition components, the temperature and pressure in dataset B were defined only for 64,945 chemical transformations (75,726 reactions), the list of catalysts was bigger, and reagents were represented as compounds (see more information in Section 3.3.3), contrary to the general classes used in dataset S.

### 3.2. Training and Test Sets

The test set for dataset S was created by randomly selecting the chemical transformations with a single reported condition. The target was to have approximately 15% of reactions in the test set. Thus, the training set of dataset S included 25,917 transformations (27,689 reactions), and the test set contained 3692 chemical transformations (3692 reactions).

For dataset B, the test set was supposed to have 20% of the reaction content and was selected from the conditions where temperature and pressure were explicitly recorded, and a single combination of conditions was reported for a transformation. As a result, the training set of dataset B consisted of 123,328 transformations (157,051 reactions); the test set included 39,261 chemical transformations (39,261 reactions).

For both datasets, since the selection was made from transformations having only one reported condition, the test set comprised only novel reactant–product pairs that were not present in the training set. The Reaxys^®^ ID corresponding to the test set of dataset B can be accessed at https://github.com/cimm-kzn/rc_cvae/tree/main/src/rc_cvae (accessed on 17 December 2025).

### 3.3. Descriptors

#### 3.3.1. Descriptors of Chemical Transformations

Chemical transformations were encoded using the Condensed Graph of Reaction approach (CGR) [25]. The CGR approach represents each reaction as a single 2D graph, a pseudomolecule containing both conventional chemical bonds and so-called dynamic bonds characterizing changed/broken/formed chemical bonds [25], as well as labels on atoms that changed their formal charge (dynamic atoms) [23]. Thus, CGR represents the whole reaction as a single graph resembling a molecular graph with additional labels. The CGRtools library was used to generate CGRs [23]. ISIDA fragment descriptors of CGR were computed using the ISIDA Fragmentor 2017 [25] software wrapped by an in-house Python CIMtools library (version 3.1.0) [26]. The fragments represent subgraphs of different topologies and sizes, and the counts of the fragments were used as descriptor values. In this study, atom-centered fragments were based on sequences of atoms and bonds of fixed length ranging from 2 to 4, with the additional option to show atom formal charges in fragment specification. More than 144,000 and 317,000 fragments were generated for training subsets of the datasets S and B, respectively. Consequently, Incremental Principal Component Analysis [27] implemented in scikit-learn [28] was applied. Latent descriptor space corresponding to the explained variance of approximately 95% was left. Details of the datasets are presented in Table 3.

#### 3.3.2. Descriptors of Conditions for Dataset S

**Figure 6:**
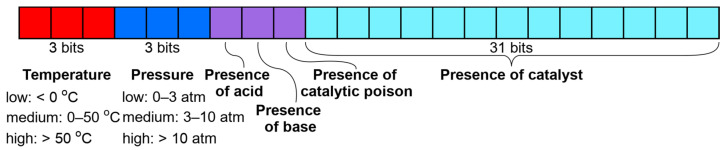
Condition vector for dataset S.

The condition vector for dataset S contained 40 bits, as described in the publication [17]—3 bits for temperature and pressure (low, medium, and high), 3 bits encoding the presence of additives (acid, base, and catalytic poison), and 31 bits for used catalysts (Figure 6). Only catalysts used in more than 100 reactions in the training set were considered. The complete list of catalysts and the definition of the temperature and pressure values range can be found in the Supplementary Materials, Table S3. The 10 most frequently occurring conditions are displayed in the Supplementary Materials (Figure S3). A combination of 1 bit out of 3 selected for temperature and pressure, 1 selected catalyst, and all possible selections of additives gives rise to 2232 possible conditions.

The primary objective of this work was to overcome the limitations of the previously developed condition ranking model (LRM) [10] in handling large condition spaces by introducing a VAE-based condition generation approach. To ensure a rigorous and fair comparison with the LRM, the same dataset and an identical reaction condition representation were used, which, in the article [10], did not include solvent information.

#### 3.3.3. Descriptors of Conditions for Dataset B

**Figure 7:**
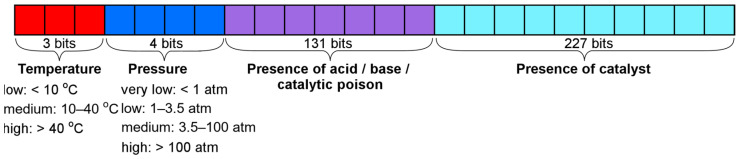
Condition vector for dataset B.

The condition vector for dataset B included 365 bits: 227 bits were allocated for the most popular catalysts, and 131 bits were reserved for all other additives. To preserve the possibility of easy comparison with the LRM [10] and the model fitted on the dataset S, the additives in the condition vector include only compounds whose roles after curation corresponded to acid, base, or catalytic poison (Figure 7). One bit here represented the presence of a certain compound used as an additive in the training set 572. Unlike dataset S, which required additive aggregation due to sparse observations, dataset B allows additives to be encoded as individual chemical compounds, owing to its larger and more diverse data coverage.

The temperature data was divided into three ranges: less than 10 °C—cooling, between 10 °C and 40 °C—ambient temperature, and more than 40 °C—heating. The pressure was categorized into four ranges: less than 1 atm—low pressure, between 1 and 3.5 atm—ambient pressure, between 3.5 and 100 atm—elevated pressure, and more than 100 atm—high pressure. These category boundaries were defined based on the statistical distribution of the training data. The 1 atm threshold corresponds to the standard atmospheric pressure. The 3.5 atm boundary marks a sharp drop in data density (with the majority of data, 64.8%, concentrated below this value). The 100 atm threshold represents a practical limit for standard high-pressure laboratory equipment. A detailed visualization of the pressure distribution confirming these thresholds is provided in the Supplementary Materials (Figures S4–S6). Unlike dataset S, dataset B contains a substantial number of reactions with missing temperature and/or pressure values. These components were marked as unknown in the data preprocessing stage.

The 10 most frequent conditions in the training set are shown in the Supplementary Materials (Figures S1 and S2), and the complete list of catalysts and additives corresponding to the condition vector is available in the Supplementary Materials (Table S4). In predicted conditions, only 1 bit corresponding to temperature and pressure can be selected, while any combination of catalysts and additives is allowed, which gives rise to astronomic condition space reaching 7 × 10^42^ potential conditions.

### 3.4. Methods

#### 3.4.1. Conditional Variational AutoEncoder

The predictive model used in this study is based on the Autoencoder (AE) architecture [20], a neural network framework that involves the compression of input data into a latent representation, which is then reconstructed during the decoding process. Although AEs have been employed in various chemoinformatics tasks [29,30,31], their latent space lacks the requisite structure for generative applications. To address this limitation, a variational autoencoder (VAE) [20] is employed, which imposes constraints on the latent space by learning a probabilistic distribution. This approach enables the generation of new data through sampling from the prior distribution.

In the VAE framework, a latent variable model is applied, where $z\in R^{M}$ represents the latent variables, **x** is a vector of D descriptors, and $p_{\phi }\left(x,z\right)$ denotes a parameterized model of the joint distribution (ϕ—weights of the decoder). The primary objective is to optimize the log-likelihood of the data, $log\int p_{\phi }\left(x,z\right)dz$. This optimization problem can be addressed by maximizing the Evidence Lower Bound (ELBO) as shown in Equation (1):

(1) $$log\int p_{\phi }\left(x,z\right)dz\geq E_{q\left(z\right)}\left[log\left(p_{\phi }\left(x|z\right)\right)\right]-KL\left(q\left(z\right)||p(z)\right),$$

where p(**z**) represents the prior distribution over the latent variable **z**, and q(**z**) is the approximate posterior distribution belonging to a family Q; KL denotes Kullback–Leibler Divergence.

Since the conditions for organic reactions must account for the reaction transformations, a modification of the VAE known as the Conditional VAE (CVAE) [20,21] is employed. Consequently, the problem of predicting reaction conditions is formulated as sampling from a generative random distribution in this study, conditioned by reaction transformation descriptors. The CVAE model is trained to learn the parameters so that the distribution becomes like a prior distribution and maximizes the log-likelihood of the training dataset using a set of chemical transformations and their conditions. This formulation allows the model to generate diverse reaction conditions tailored to the given transformation, thereby circumventing the necessity for an exhaustive enumeration of potential conditions. An approach that is similar but not entirely the same was proposed in publications [9,32].

**Latent space distributions**. Three latent space distributions were investigated to assess their impact on model performance. The baseline model employed a standard Gaussian distribution (g-CVAE), with the posterior distribution q(**z**) parameterized by mean $\mu \in R^{m}\;$ and standard deviation $\sigma \in R^{m}$ (Equation (2)) [9,29]. This distribution is widely employed in VAE frameworks [20,21]:

(2) $$q\left(z|\mu ,\sigma \right)=N\left(\mu ,\sigma^{2}\right),$$

To reflect the non-linear structure of the data, which cannot fit a standard Gaussian distribution, the Riemannian Normalizing Flow distribution (rnf-CVAE) was incorporated. Its posterior distribution q(**z**) is parameterized by the mean $\mu \in R^{m}$, the standard deviation $\sigma \in R^{m}$, and the Jacobian determinant of the inverse flow transformation (Equation (3)). This approach follows the method proposed by Wang et al. [33]:

(3) $$q\left(z|\mu ,\sigma \right)=N\left(\mu ,\sigma^{2}\right)\left|det\frac{\partial f^{-1}}{\partial z}\right|,$$

where f is a learnable function that transforms the latent variable **z** from the base distribution (like Gaussian) to a more complex one. This transformation is parameterized and learned during training, allowing the model to adapt the posterior distribution q(**z**) to better match the data structure.

Additionally, a hyperspherical uniform distribution (h-CVAE) was tested, following the approach of Davidson et al. [34], where the latent space is confined to a uniform distribution over a hypersphere to promote anisotropy and to capture non-Gaussian distribution in another way, thus enhancing the model’s generative quality. The von Mises–Fisher (vMF) distribution, often referred to as the Normal Gaussian distribution on a hypersphere, was used for this purpose [35]. Its posterior distribution is parameterized by $\mu \in R^{m}$, and $k\in R_{\geq 0}$, which controls the concentration around **μ**. When **k** = 0, the vMF simplifies to a uniform distribution (Equation (4)):

(4) $$q\left(z|\mu ,k\right)=C_{m}\left(k\right)e^{k\mu^{T}z},$$

$$\mathrm{where}\;C_{m}\left(k\right)=\frac{k^{\frac{m}{2}-1}}{{\left(2\pi \right)}^{\frac{m}{2}}I_{\frac{m}{2}-1}\left(k\right)},$$

where $I_{v}$ denotes the modified Bessel function of the first kind at order v.

**Figure 8:**
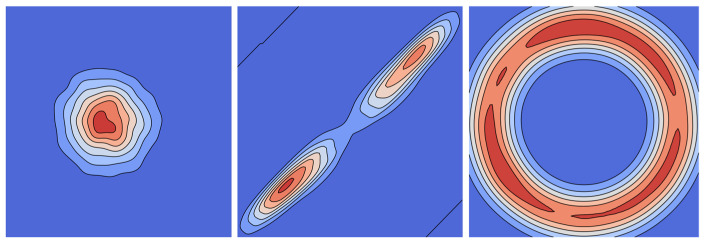
Visualization of Gaussian (**left**), Riemannian Normalizing Flow (**center**), and hyperspherical uniform kernel (**right**) distributions for 2D space using Gaussian kernel density estimation.

The three distributions (illustrated in Figure 8) were evaluated under identical training conditions to determine the optimal latent space formulation for predicting reaction conditions.

**Reparameterization trick**. The reparameterization trick plays a critical role in training VAEs, enabling efficient gradient-based optimization of the variational lower bound. In VAEs, latent variables (**z**) are typically sampled from a learned posterior distribution $q_{\psi }\left(z|x\right)$, parameterized by a neural network (where ψ represents the weights of the encoder). This sampling introduces stochasticity, which obstructs backpropagation due to the non-differentiable nature of direct sampling. To address this issue, the reparameterization trick decouples the randomness from the model parameters by expressing **z** as a deterministic function of the distribution parameters and an auxiliary noise variable ϵ. Specifically, the latent variable **z** for the g-CVAE is reparametrized as follows (Equation (5)):

(5) z ~ μ+σ×ϵ,ϵ ~ N(0,I),

where N(0,I)  represents the multivariate Gaussian distribution with a mean vector of zeros (**0**) and a covariance matrix that is the identity matrix (**I**).

Originally proposed by Kingma and Welling [20] and Rezende et al. [36], the reparameterization trick was later extended by Davidson et al. [34] to accommodate the von Mises–Fisher distribution, thereby broadening its applicability to non-Gaussian latent spaces. For rnf-CVAE, the reparameterization process (Equation (6)) remains fundamentally the same, as described in Equation (5):

(6) $$z_{0}\;\sim \;\mu +\sigma ⨀\epsilon ,\epsilon \;\sim \;N\left(0,I\right),$$

However, the rnf-CVAE incorporates a learnable transformation function f applied iteratively through normalizing flows (Equation (7)):

(7) $$z_{n}\;\sim \;f_{n}\circ f_{n-1}\circ \ldots \circ f_{1}\left(z_{0}\right),$$

where n represents the number of normalizing flow steps. This iterative application of transformations allows for more flexible modeling of complex distributions in the latent space.

To enforce the hyperspherical latent space (for h-CVAE), a more complex matrix multiplication is required, as described by Davidson et al. [34]. The latent variables (Equation (8)) are obtained by transforming two auxiliary random variables: $v^{\prime }$ drawn from the multivariate Gaussian distribution and ε drawn from a Beta distribution. The latter requires an acceptance–rejection procedure, which introduces additional constraints on admissible values of ε. For clarity, these constraints are not explicitly included in the generalized expression above; the detailed algorithm and sampling procedure are provided in Appendix A of Davidson et al. [34]:

(8) $$z=\left(I_{m}-2\frac{uu^{T}}{{\left\|u\right\|}^{2}}\right){\bar{a}}^{T},$$

$$\mathrm{where}\;\bar{a}=\left(\frac{1-\left(1+b\right)\varepsilon }{1-\left(1-b\right)\varepsilon },\;\sqrt{1-{\left(\frac{1-\left(1+b\right)\varepsilon }{1-\left(1-b\right)\varepsilon }\right)}^{2}}\cdot \frac{v^{\prime }}{\left\|v^{\prime }\right\|}\right),$$

$$u={\left[1,0,\ldots ,0\right]}^{T}-\mu ,$$

$$v^{\prime }\;\sim \;N\left(0,I_{m-1}\right),$$

$$\varepsilon \;\sim \;Beta\left(\frac{m-1}{2};\frac{m-1}{2}\right),$$

$$b=\frac{-2k+\sqrt{4k^{2}+{\left(m-1\right)}^{2}}}{m-1},$$

where $I_{m}$ is the m × m identity matrix; m denotes the dimensionality of the hypersphere; k represents the concentration parameter for the vMF distribution.

**Figure 9:**
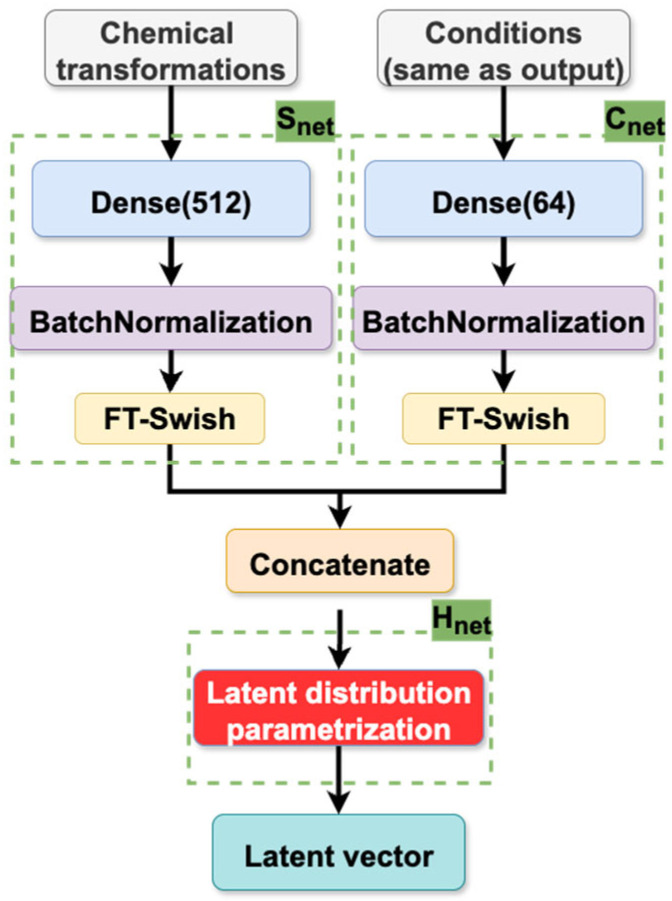
Encoder of the CVAE models trained on datasets S and B.

**Figure 10:**
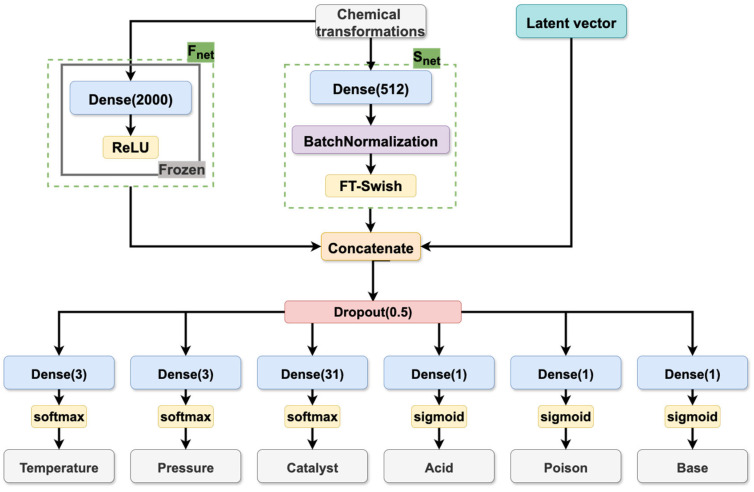
Decoder of the CVAE model trained on dataset S.

**Figure 11:**
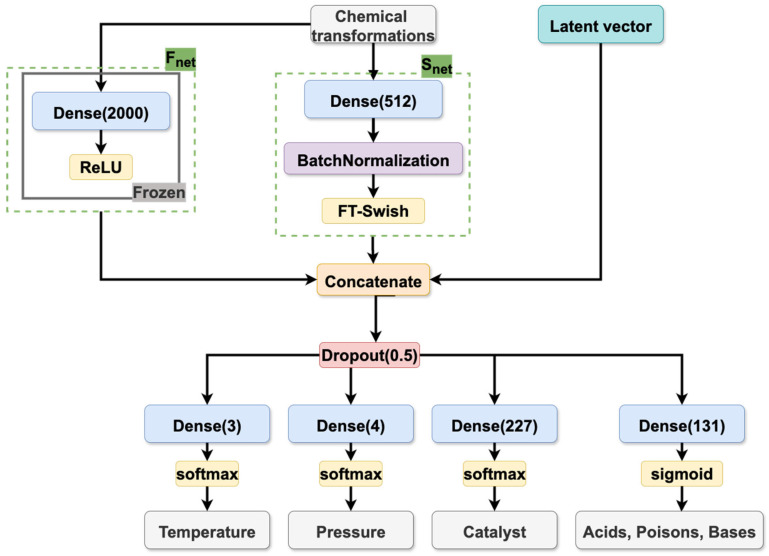
Decoder of the CVAE model trained on dataset B.

**Model architecture**. The architecture of the CVAE model is composed of two primary components: the encoder and the decoder. The function of the encoder is to map input reaction conditions and chemical transformation descriptors into the latent space, while the decoder is responsible for the reconstruction of conditions by sampling from this distribution. The architectural details of the encoder and decoder are illustrated in Figure 9, Figure 10 and Figure 11, which have been specifically designed for reaction condition prediction for datasets S and B, respectively.

As illustrated in Figure 9, the encoder passes reaction conditions through a “reaction condition” feed-forward neural network (C_net_) comprising a single hidden layer of 64 neurons with a Flatten-T Swish activation function [37]. Simultaneously, CGR-based fragment descriptors of the chemical transformations (described in Section 3.3.1) are processed by a separate “reaction transformation” feed-forward neural network (S_net_) containing a single hidden layer with 512 neurons with Flatten-T Swish activation. The outputs from these networks are then concatenated and passed to the H_net_ layer, which parameterizes the latent distribution and defines mean and variance for the Gaussian and Riemannian Normalizing Flow distribution; mean and concentration around the mean for the hyperspherical distribution. The latent vectors were sampled from the corresponding distribution following the reparameterization trick (as described above).

The decoder (see Figure 10 and Figure 11) receives the latent variables and chemical transformation embedding vectors and generates the condition components through multiple output heads. The embedding of the chemical transformations is generated by two parallel networks, S_net_ (as described above) and a pre-trained model (F_net_), whose architecture was taken from the Likelihood Ranking Model (LRM) described in [10]. The incorporation of the latter was found to be beneficial for the model quality. The F_net_ processes CGR-based fragment descriptors of the chemical transformation through a feed-forward neural network with a single hidden layer of 2000 neurons and a ReLU activation function. The outputs of both the F_net_ and the S_net_ capture structural information about the transformation, thereby enriching the latent space representation with chemically relevant features.

**Loss functions**. Multiple loss functions were utilized to enhance the performance of the CVAE model, reflecting the distinct characteristics of each condition component. Overall loss can be expressed as Equation (9):

(9) L=FL(catalyst)+FL(additives)+EMD(temperature)+EMD(pressure)+β·KL(distribution),

where *β* is the regulating constant. Each component corresponds to a specific aspect of the loss function, which will be described below.

**Catalyst prediction**. Since both datasets S and B implied that only one catalyst bit is activated (one-hot encoding), the Softmax output function was used in the catalyst prediction head. Catalyst prediction was performed using Extended Softmax Focal Loss (FL, Equation (10)), a variant of cross-entropy loss, designed to mitigate class imbalance by focusing on hard-to-classify samples:

(10) $$FL\left(p\right)=\sum_{i=1}^{N}-\alpha \cdot {\left(1-p_{i}\right)}^{\gamma }{\cdot y}_{i}\cdot log(p_{i}),$$

where $p_{i}\;$ is the predicted probability of the ith class; $y_{i}$ denotes the actual bit label for the ith element of bitstring; N is the number of classes. The weighting factor, α, is employed to balance the importance of different classes, particularly in cases where class distribution is highly imbalanced. The value of α is typically constrained to the interval [0, 1]. The parameter, γ, serves to adjust the rate at which easy examples are down-weighted. A higher γ value emphasizes hard-to-classify examples more (γ ∈ R). In this study, γ = 2 and α = 0.25 were selected, which were the values employed in the original publication [38].

**Additive prediction**. Multi-hot encoding of additives was used in datasets S and B. Therefore, the binary cross-entropy function was employed for dataset S (BL, Equation (11)), whereas the binary Focal Loss [38] (Equation (12)) was utilized for dataset B. It is noteworthy that the CVAE models trained on dataset S were capable of predicting the presence or absence of certain classes of additives (acid/base/catalytic poison). In contrast, dataset B did not involve the categorization of additives into distinct classes but rather every bit corresponded to a certain compound that might serve distinct roles, such as acting as an acid or a solvent, depending on the reaction context (e.g., acetic acid may be used as a solvent in some reactions, whereas, in other reactions, it might be used for catalyst activation):

(11) $$BL\left(p\right)=-y_{i}\cdot log\left(p_{i}\right),$$

$$\mathrm{where}\;p_{i}=\{\begin{array}{c} p\;\;\;\;\;\;\;\;\;\;\;\mathrm{if}\;y=1;\\ 1-p\;\;\;\;\;\;\mathrm{otherwise}, \end{array}$$

(12) $$FL\left(p_{i}\right)=-\alpha_{i}\cdot {\left(1-p_{i}\right)}^{\gamma }\cdot log(p_{i})$$

$$\mathrm{where}\;p_{i}=\{\begin{array}{c} p\;\;\;\;\;\;\;\;\;\;\mathrm{if}\;y=1;\\ 1-p\;\;\;\;\;\;\mathrm{otherwise}, \end{array}$$

$$\alpha_{i}=\{\begin{array}{c} \alpha \;\;\;\;\;\;\;\;\;\;\;\mathrm{if}\;y=1;\\ 1-\alpha \;\;\;\;\;\;\mathrm{otherwise}, \end{array}$$

where α ∈ [0, 1], γ ∈ R. In this study, γ = 2 and α = 0.25 were selected [38].

**Temperature and pressure prediction.** Ranges of temperature and pressure were treated as ordered categorical variables and optimized using Earth Mover’s Distance (EMD) loss [39,40] (Equation (13)), which penalizes misclassifications proportionally to the class distance [39]. For example, if the actual value for pressure is “low”, the model is penalized harder for predicting “high” pressure than for predicting “medium” pressure and is not penalized for high confidence prediction of “low” pressure class. The EMD loss function is expressed as follows:

(13) $$EMD\left(y,p\right)={\left(\frac{1}{K}\sum_{k=1}^{K}{\left|{CDF}_{y}\left(k\right)-{CDF}_{p}\left(k\right)\right|}^{r}\right)}^{\frac{1}{r}},$$

where CDF_y_(k), CDF_p_(k) denote the cumulative distribution functions for the true (**y**) and predicted (**p**) distributions, respectively, at the kth class. The CDF is defined as the sum of the probabilities of class k, providing a smooth, non-decreasing function that represents the distribution. The parameter r denotes the type of distance metric employed; K signifies the number of classes in the distribution. In this study, r = 2 was employed, signifying the implementation of the Euclidean distance (L2 norm between the CDFs), thereby facilitating the gradient descent optimization process. This metric is employed to evaluate the extent to which the predicted distribution aligns with the actual distribution by comparing the cumulative probabilities across all classes.

It should be noted that for records with missing temperature or pressure values in dataset B, the corresponding decoder output heads were excluded from the loss calculation, and no gradients were propagated through them. This allowed the model to learn effectively from the available condition components (e.g., catalysts and additives) without introducing bias due to missing data.

**Implementation details.** To learn the prior latent distribution of CVAE, the Kullback–Leibler Divergence (KL loss) was used for both g-CVAE and h-CVAE models. In both g-CVAE and h-CVAE models, β was set to 0.001 as a multiplier for the KL loss to prevent model collapse—a phenomenon in which the VAE generates only the most frequent option from the training data. In the rnf-CVAE configuration, Maximum Mean Discrepancy (MMD) loss [41] was combined with KL loss to enhance the distributional alignment of the latent space [33]. For rnf-CVAE, β was gradually varied from 0 to 0.8 during training. Additionally, a special penalty loss was introduced for rnf-CVAE to ensure the proper learning of flows, as described in the original method [33]. No dedicated hyperparameter tuning was conducted for the models in this study. Instead, hyperparameters were selected based on established recommendations from the literature [33,38,41].

The implementation of the models was conducted within the TensorFlow framework [42] and utilized the Adam optimizer [43] for training. The initial learning rate was set to 0.001, which was reduced to 0.00025 over 200 epochs. The latent space dimensionality was fixed at 32, which was identified during preliminary experiments as the largest dimension that remained numerically stable for all three CVAE variants. For the rnf-CVAE model, 3 normalizing flows were used. A batch size of 128 was utilized for all experiments, and training was terminated after 200 epochs.

Official implementations of h-CVAE [34], originally developed in TensorFlow 1, were adapted for TensorFlow 2 and are available at the following link: https://github.com/cimm-kzn/s-vae-tf (accessed on 17 December 2025). The source code for all three CVAE models (g-CVAE, rnf-CVAE, and h-CVAE) is publicly available at https://github.com/cimm-kzn/rc_cvae (accessed on 17 December 2025).

#### 3.4.2. Other Models

To benchmark the performance of g-CVAE, rnf-CVAE, and h-CVAE models, a comparative analysis was conducted against several existing approaches for reaction condition prediction. These models include both classical machine learning techniques and state-of-the-art deep learning frameworks:

1. Null model predicts RCs by selecting the most frequently occurring combination of condition components from the training set. However, it should be noted that the training set of dataset B includes some records with unknown temperature or pressure (see Supplementary Materials, Figures S1 and S2); therefore, these conditions were ignored by the model during the definition of a metric of the model. This model serves as a baseline, reflecting the probability of correct predictions based solely on the frequency distribution of conditions in the dataset.
2. The ranking k Nearest Neighbors (kNN) method [10] is employed to retrieve RCs from the training set that are most similar to the query transformation. The assessment of reaction similarity was conducted through the calculation of the Euclidean distance, utilizing CGR-based fragment descriptors that were compressed with the assistance of Incremental Principal Component Analysis [27], as described in Section 3.3.1.
3. A Likelihood Ranking Model (LRM) [10] applies to a feed-forward neural network with a single hidden layer of 2000 neurons and a ReLU activation function to predict reaction conditions. The output layer has sigmoid activation and predicts each condition component’s probabilities and ranks them according to likelihood [10].
4. The model from ASKCOS proposed by Gao H. et al. [6] is available on GitHub (version 1.0, commit ec2a858) https://github.com/Coughy1991/Reaction_condition_recommendation (accessed on 13 March 2019). and predicts the catalyst, first solvent, second solvent, first reagent, second reagent, and temperature as a continuous value. The predicted temperature value was then mapped to the corresponding range based on the criteria outlined above (see Section 3.3.2 and Section 3.3.3). The default option is to enumerate 18 conditions based on the best two catalysts, the first three solvents, one second solvent, the first three reagents, and one second reagent. Given that the output of the ASKCOS model is represented by the Reaxys^®^ chemical ID, SMILES, or compound name, the name standardization procedure is required. The complete match of the combination of conditions was checked; in our assessments, the pressure was always considered to be correctly predicted because the ASKCOS model proposed by Gao et al. does not predict it. Predicted solvents were ignored as proposed models do not predict solvents. So, the provided values were somehow biased toward the overestimation of ASKCOS model performance. Nonetheless, it is worth mentioning that the ASKCOS model was trained on the whole reaction dataset and, unlike the other models, was not specifically tailored to the hydrogenation reaction case, which likely explains its lower predictive performance in this study.

### 3.5. Evaluation Metric

The performance of the CVAE models and comparative approaches was assessed using the “precision at k” (p@k) metric. This metric is a rank-based evaluation method commonly employed in reaction condition prediction tasks. Precision at k quantifies the percentage of test set reactions for which at least one experimentally recorded combination of RCs appears within the top-k predicted results (Equation (14)). A prediction is considered correct only if it exactly matches the experimentally recorded combination of conditions; any deviation is classified as incorrect. Various combinations of additives (e.g., catalyst poison, acid, or base) are permitted, except when both an acid and a base are present simultaneously. The test sets for datasets were constructed exclusively from reactions for which temperature and pressure were explicitly recorded. In some cases, no additives may be present, with only temperature, pressure, and catalyst specified. It is important to note that solvent prediction is not included in this study:

(14) $$p@k=\frac{n@k}{N}\cdot 100\%$$

where n@k is the number of reactions for which at least one correct combination of conditions is found within the top-k predictions, and N is the total number of reactions in the test set.

The g-CVAE, rnf-CVAE, and h-CVAE models were sampled 5000 times per reaction to ensure a comprehensive evaluation of generative model predictions. The predicted condition sets were then ranked based on their frequency of occurrence, and the top-k predictions were selected for precision calculation.

Using p@k reflects the practical relevance of condition prediction models, as multiple condition combinations can yield successful chemical transformations. A higher p@k value indicates the model’s ability to consistently generate valid reaction conditions within a limited number of guesses, mirroring real-world scenarios in chemical synthesis planning.

It is important to note that the reported p@k values represent point estimates derived from this single test set. While these metrics provide a direct quantitative comparison of the models, a formal statistical analysis [44] to test the significance of the observed differences is impossible to perform due to time-consuming model training and inference, which takes up to a few days.

## 4. Conclusions

In this study, we have proposed, benchmarked, and validated using the conditional variational autoencoder (CVAE) generative models for predicting sets of reaction conditions for hydrogenation reactions and other H_2_-mediated reactions. The efficacy of the CVAE models in predicting a wide range of RCs has been demonstrated through sampling from the prior random distribution, thereby allowing the models to generate diverse combinations of catalysts, additives (acids, bases, and catalytic poisons), temperatures, and pressures without requiring exhaustive enumeration. Multiple predictions for a query reaction were obtained through repeated sampling from the distribution. In contrast to traditional models that predict individual condition components, the CVAE models explicitly generate RCs. This integrated prediction framework enhances the consistency between condition components, thereby reducing the risk of incompatible combinations.

Using two datasets of different scales and complexities extracted from the Reaxys^®^ database, we showed that the CVAE models consistently outperform state-of-the-art models (kNN, LRM, and the model from ASKCOS) in retrieving experimentally recorded reaction conditions. On the smaller dataset S, where full enumeration of all condition combinations is possible, the CVAE models achieved performance comparable to the LRM. On the substantially larger and more diverse dataset B, where enumeration is infeasible, the CVAE models, particularly the h-CVAE, demonstrated clear improvements over other models across all top-k metrics, highlighting their suitability for large-scale reaction condition prediction.

Among the tested latent space formulations (standard Gaussian distribution, Riemannian Normalizing Flow, and hyperspherical uniform), the h-CVAE exhibited the most stable and robust behavior. By enforcing a hyperspherical uniform, the h-CVAE promotes a more even distribution of latent representations and mitigates issues such as latent space collapse. This characteristic enables the model to capture not only frequently observed but also rare reaction conditions, improving its generalization to unseen data. This ability to predict rare conditions is especially valuable for complex reactions, where diverse pathways can lead to successful chemical transformations. Although this approach introduces additional computational cost, its respective strengths—enhanced expressiveness and uniformity in latent space—provide significant performance gains over the conventional g-CVAE. These advancements underscore the potential of sophisticated latent space modeling techniques for developing powerful and efficient tools for reaction condition prediction, ultimately broadening the scope and reliability of chemical synthesis planning.

In future work, the predictive accuracy and robustness of the proposed CVAE models may be further enhanced by exploring alternative model architectures. A promising direction is reformulating reaction condition prediction as an autoregressive generation task using a transformer decoder. Such an approach would allow the model to generate individual components of the reaction conditions sequentially, thereby promoting greater internal consistency and ensuring compatibility between catalysts, additives, temperatures, and pressures. This modification may help reduce inconsistencies in multi-component predictions and enhance the model’s generalization across chemically diverse datasets. In addition, applying the validated CVAE framework to more recent and expanded reaction datasets represents an important next step toward improving its practical relevance and applicability.

The code base for the proposed models is available at https://github.com/cimm-kzn/rc_cvae (accessed on 17 December 2025). Models cannot be published as proprietary data were used for modeling.

## Figures and Tables

**Table 1:** Top-k (where k = 1, 3, or 10) results of catalyst (c@k), additives ^1^ (ad@k), range of temperature (t@k), and pressure (p@k) on dataset S.

No.	Precision at k	Models		
		g-CVAE	rnf-CVAE	h-CVAE
1	c@1	79.04	80.17	79.39
2	c@3	87.11	87.89	86.94
3	c@10	93.77	93.77	93.77
4	ad@1	80.39	85.37	83.83
5	ad@3	90.25	93.12	93.15
6	ad@10	96.45	97.02	97.62
7	t@1	91.68	92.44	92.77
8	t@3	94.88	95.29	95.29
9	t@10	98.51	98.21	98.02
10	p@1	80.01	80.82	81.18
11	p@3	95.23	92.42	93.91
12	p@10	99.67	97.91	99.13

^1^ Only catalytic poison, acid, or base.

**Table 2:** Top-k (k = 1, 3, or 10) results of catalyst (c@k), additives ^1^ (ad@k), range of temperature (t@k), and pressure (p@k) on dataset B.

No.	Precision at k	Models		
		g-CVAE	rnf-CVAE	h-CVAE
1	c@1	69.43	70.57	69.27
2	c@3	79.93	83.41	79.23
3	c@10	84.7	90.08	91.55
4	ad@1	79.84	80.85	80.04
5	ad@3	80.61	84.82	82.92
6	ad@10	81.52	88.54	90.7
7	t@1	82.34	83.19	82.46
8	t@3	88.93	89.11	92.18
9	t@10	92.06	93.08	98.88
10	p@1	73.25	75.08	73.29
11	p@3	89.48	84.46	95.72
12	p@10	91.87	89.82	97.04

^1^ Only catalytic poison, acid, or base.

**Table 3:** Overall data statistics.

No.		Datasets	
		S	B
1	Training set size (reactions)	27,689	157,051
2	Test set size (reactions)	3692	39,261
3	Descriptor space (PCA)	500	1000
4	Output dimension	40	365
5	Potential ^1^ condition space	2232	~7 × 10^42^
6	Number of conditions used in the training set	477	3355

^1^ Number of conditions that can potentially be generated for the given output representation.

## Data Availability

Because the Reaxys^®^ database is commercially available, we do not have permission to release the datasets to the public.

## Supplementary Materials

The following supporting information can be downloaded at https://www.mdpi.com/article/10.3390/molecules31010075/s1, Figure S1: Top-10 most frequent conditions in the training set for dataset B. Conditions with unknown temperature or pressure were ignored (temperature: low—less 0 °C, medium—0–40 °C, and high—more 40 °C; pressure: very low—less 1 atm, low—1–3.5 atm, medium—3.5–100 atm, and high—more 100 atm); Figure S2: Top-10 most frequent conditions in the training set for dataset B. Conditions with unknown temperature or pressure were not ignored. (temperature: low—less 0 °C, medium—0–40 °C, and high—more 40 °C. Pressure: very low—less 1 atm, low—1–3.5 atm, medium—3.5–100 atm, and high—more 100 atm); Table S1: Example of h-CVAE model predictions. Table S2: Example of model predictions compared with recorded context test set where the h-CVAE model correctly identified the combination of RCs, with the exception of one condition (n/p—not predicted solvents); Table S3: List of the most common catalysts in the training set of dataset S; Figure S3: Top-10 most frequent conditions in the training set for dataset S (temperature: low—less 0 °C, medium—0–50 °C, and high—more 50 °C; pressure: low—0–3 atm, medium—3–10 atm, and high—more 10 atm); Table S4: List of the reagents in the training set of dataset B; Figure S4: Distribution of pressure values in the training dataset B (detailed view: 0–15 atm range). The histogram (bin size: 0.5 atm) highlights the extreme data density in the 1–3.5 atm range, which contains 64.8% of all data. The sharp drop in frequency beyond 3.5 atm justifies its selection as a category threshold. Vertical dashed lines mark the 1 atm (standard atmospheric pressure) and 3.5 atm (empirical density drop) boundaries; Figure S5: Complete distribution of pressure values in the training dataset B (overview: 0–120 atm range). The histogram uses variable-width bins to accurately represent the four defined pressure categories: very low (<1 atm, blue), low (1–3.5 atm, green), medium (3.5–100 atm, orange), and high (≥100 atm, red). Numbers above bars (shown for bins containing >1% of the data) indicate the percentage of total data in each 5-atm interval. The plot visually underscores the sharp decline in data density beyond 3.5 atm and the practical 100 atm limit for standard laboratory equipment; Figure S6: Cumulative distribution function (CDF) of pressure in the training dataset B. The curve shows the cumulative percentage of data with pressure below each value (i.e., P(pressure < x)), providing a statistical justification for the chosen category boundaries. Key thresholds are annotated: <1 atm (1.4% of data), <3.5 atm (65.6% of data), and <100 atm (98.3% of data). The 3.5 atm threshold approximates the point where ~75% of data have lower pressures, marking the transition from high-density to low-density data regions.

## Abbreviations

The following abbreviations are used in this manuscript:

RC	Reaction conditions
AE	Autoencoder
VAE	Variational autoencoder
CASP	Computer-aided synthesis planning
CDF	Cumulative distribution function
CGR	Condensed graph of reaction
EMD	Earth Mover’s Distance
ELBO	Evidence Lower Bound
FL	Focal loss
g-CVAE	Gaussian conditional variational autoencoder
h-CVAE	Hyperspherical conditional variational autoencoder
rnf-CVAE	Riemannian Normalizing Flow conditional variational autoencoder
HTE	High-throughput experimentation
kNN	k Nearest Neighbors
KL	Kullback–Leibler Divergence
LRM	Likelihood Ranking Model
MMD	Maximum Mean Discrepancy
PCA	Principal Component Analysis
p@k	Precision at k
ReLU	Rectified Linear Unit
vMF	von Mises–Fisher Distribution

## Appendix A

### Appendix A.1. Structure Standardization of Dataset B

More than 264 thousand reactions of catalytic hydrogenation and 402 thousand condition records were used in this research as the dataset B. These reactions have been retrieved from the Reaxys^®^ database in 2017 by the Reaxys^®^ API using the following query:

- Gaseous hydrogen as a reagent;
- The number of stages is equal to 1;
- There are not the keyword “steps”.

The last two parts of the query allow us to collect only one-step hydrogenation reactions.

Because some structures were not standardized (benzyl rings are not aromatized, explicit hydrogens are included, some groups, such as nitro group or sulfoxide group, are not transformed to a unique representation, etc.), the first stage was structure standardization.

Structure standardization was carried out using the in-house CGRtools (version 3.1) library [26]. Standardization includes several steps. Firstly, all explicit hydrogens were removed; functional group standardization rules were applied to reactants and products. All molecules were checked for the presence of radicals and valence errors. Moreover, it was checked that in the reaction, the number of cations corresponds to the number of anions. Information about the stereochemistry of the reaction was ignored. Reaction duplicates were removed by SMIRKS comparison. Secondly, atom-to-atom mapping was generated by using ChemAxon Standardizer [24]. An atom-to-atom mapping with the least number of dynamic atoms and bonds was chosen. Thirdly, reagents (the same molecules from the left and right parts of the reaction) were removed from every reaction. Reactions with competitive products were deleted. A reaction was considered a reaction with competitive products if the molecules of the reaction products were very similar in size (the number of different atoms is less than 4).

In-house database management system for chemical data, CGRdb (version 3.1.1) [45], was used to store standardized data.

### Appendix A.2. Condition Standardization of Dataset B

Condition standardization was an important part of dataset preparation. It includes temperature and pressure values extraction both from fields containing numerical values of temperature and pressure, and from a text field of conditions. Data from numerical fields was considered a higher priority than from text fields. Chemical agents from solvents, reagents, and catalysts fields were extracted, standardized, and their role in the hydrogenation reaction was redefined by semi-automatically adding tags (such as catalytic poison, catalyst, acid, or base). It was necessary because, firstly, the names of chemical agents in the database were “raw” and the same compound under the conditions of different reactions could be called differently—for example, Raney nickel turned out to be the absolute record holder for the number of “raw” names per standard—358 unique “raw” names. Secondly, the compounds were not always brought into the field according to their respective roles in the reaction. For example, water was found in the catalyst field in some cases. In total, 241 unique catalysts were present in the reactions of our dataset, of which 58 catalysts were found in 100 or more reactions. Standardization of names was carried out using a dictionary with the structure of “the raw name—the standard name”. This dictionary was compiled semi-automatically and covered 95.63% of the dataset. It included 3257 “raw” names (1666 “standard” names). All chemical agents’ names in the reaction were standardized in 87.31% of cases. A catalyst such as the Lindlar catalyst deserves special attention. It is a specific catalyst, characterized by a method of preparation, and in most cases, it consists of palladium deposited on calcium carbonate, which is then poisoned with various forms of lead or sulfur. We considered a Lindlar catalyst palladium deposited on substrates such as calcium carbonate, barium sulfate, barium carbonate, carbon, and poisoned with lead-containing compound and/or quinoline and/or sulfur compound [46]. The condition standardization protocol was represented in more detail in our previous paper [10], and it was illustrated in the scheme in Figure A1. It should be noted that reactions with unknown temperature and/or pressure were not deleted and used for training. The additional step of condition standardization was applying some rules to standardized reaction conditions; for example, if under the reaction conditions there is simultaneously a metal acting as a catalyst (for example, Rh) and one of the most common substrates (for example, SiO_2_), then they are combined into one catalyst—Rh/SiO_2_. Thus, such rules were applied for Rh, Ru, Pt, Pd, Ni, Ir, PtO_2_, PdO, and such substrates as C, SiO_2_, Al_2_O_3_, and BaSO_4_. Moreover, some rules were dedicated to the Lindlar catalyst. So, one restriction was applied to the dataset in the standardization process—only reactions with one and only one catalyst were kept. As a result, the dataset B included 162,590 chemical transformations, and 196,313 reactions (Figure A1); both temperature and pressure are defined for 64,945 transformations and 75,726 reactions.
