# Supplementary material for: Conditional Variational AutoEncoder to Predict Suitable Conditions for Hydrogenation Reactions
Source: Molecules. 2025 Dec 24;31(1):75. doi: 10.3390/molecules31010075 (PMC12786955; doi:10.3390/molecules31010075)
Supplement: Supplementary file 1 [file molecules-31-00075-s001.zip › molecules-3999607-supplementary.pdf]

Conditional Variational AutoEncoder to Predict Suitable Conditions for Hydrogenation Reactions

Daniyar Mazitov [1], Timur Gimadiev [1,2]*, Assima Poyezzhayeva [1,2]*, Valentina Afonina [1] and Timur Madzhidov [3]

[1] A.M. Butlerov Institute of Chemistry, Kazan Federal University, Kremlevskaya Str. 18, 420008, Kazan, Russia; daniyarttt@gmail.com (D.M.), timur.gimadiev@gmail.com (T.G.), asima8904@gmail.com (A.P.), valiaafo@yandex.ru (V.A).

[2] Federal Research Center "Kazan Scientific Center of the Russian Academy of Sciences", Lobachevskogo Str. 2/31, 420111 Kazan, Russia; timur.gimadiev@gmail.com (T.G.), asima8904@gmail.com (A.P.)

[3] Chemistry Solutions, Elsevier, London, EC2Y 5AS, UK; tmadzhidov@gmail.com (T.M.).

* Correspondences: gimadiev@gmail.com (T.G.), asima8904@gmail.com (A.P.).

Table of content:

Figure S1: Top-10 most frequent conditions in the training set for dataset B. Conditions with unknown temperature or pressure were ignored (temperature: low - less 0 °C, medium - 0-40 °C, high - more 40 °C; pressure: very low – less 1 atm, low - 1-3.5 atm, medium – 3.5-100 atm, high - more 100 atm);

Figure S2: Top-10 most frequent conditions in the training set for dataset B. Conditions with unknown temperature or pressure were not ignored. (temperature: low - less 0 °C, medium - 0-40 °C, high - more 40 °C. pressure: very low – less 1 atm, low - 1-3.5 atm, medium – 3.5-100 atm, high - more 100 atm);

Table S1: Example of h-CVAE model predictions.

Table S2: Example of model predictions compared with recorded context test set where the h-CVAE model correctly identified the combination of RC, with the exception of one condition;

Table S3: List of the most common catalysts in the training set of dataset S;

Figure S3: Top-10 most frequent conditions in the training set for dataset S (temperature: low - less 0 °C, medium - 0-50 °C, high - more 50 °C; pressure: low - 0-3 atm, medium - 3-10 atm, high - more 10 atm);

Table S4: List of the reagents in the training set of dataset B;

Figure S4: Distribution of pressure values in the training dataset B (detailed view: 0–15 atm range). The histogram (bin size: 0.5 atm) highlights the extreme data density in the 1–3.5 atm range, which contains 64.8% of all data. The sharp drop in frequency beyond 3.5 atm justifies its selection as a category threshold. Vertical dashed lines mark the 1 atm (standard atmospheric pressure) and 3.5 atm (empirical density drop) boundaries;

Figure S5: Complete distribution of pressure values in the training dataset B (overview: 0–120 atm range). The histogram uses variable-width bins to accurately represent the four defined pressure categories: very low (<1 atm, blue), low (1–3.5 atm, green), medium (3.5–100 atm, orange), and high (≥100 atm, red). Numbers above bars (shown for bins containing >1% of the data) indicate the percentage of total data in each 5-atm interval. The plot visually underscores the sharp decline in data density beyond 3.5 atm and the practical 100 atm limit for standard laboratory equipment;

Figure S6: Cumulative distribution function (CDF) of pressure in the training dataset B. The curve shows the cumulative percentage of data with pressure below each value (i.e., P(pressure < x)), providing a

statistical justification for the chosen category boundaries. Key thresholds are annotated: <1 atm (1.4% of data), <3.5 atm (65.6% of data), and <100 atm (98.3% of data). The 3.5 atm threshold approximates the point where ~75% of data have lower pressures, marking the transition from high-density to low-density data regions.

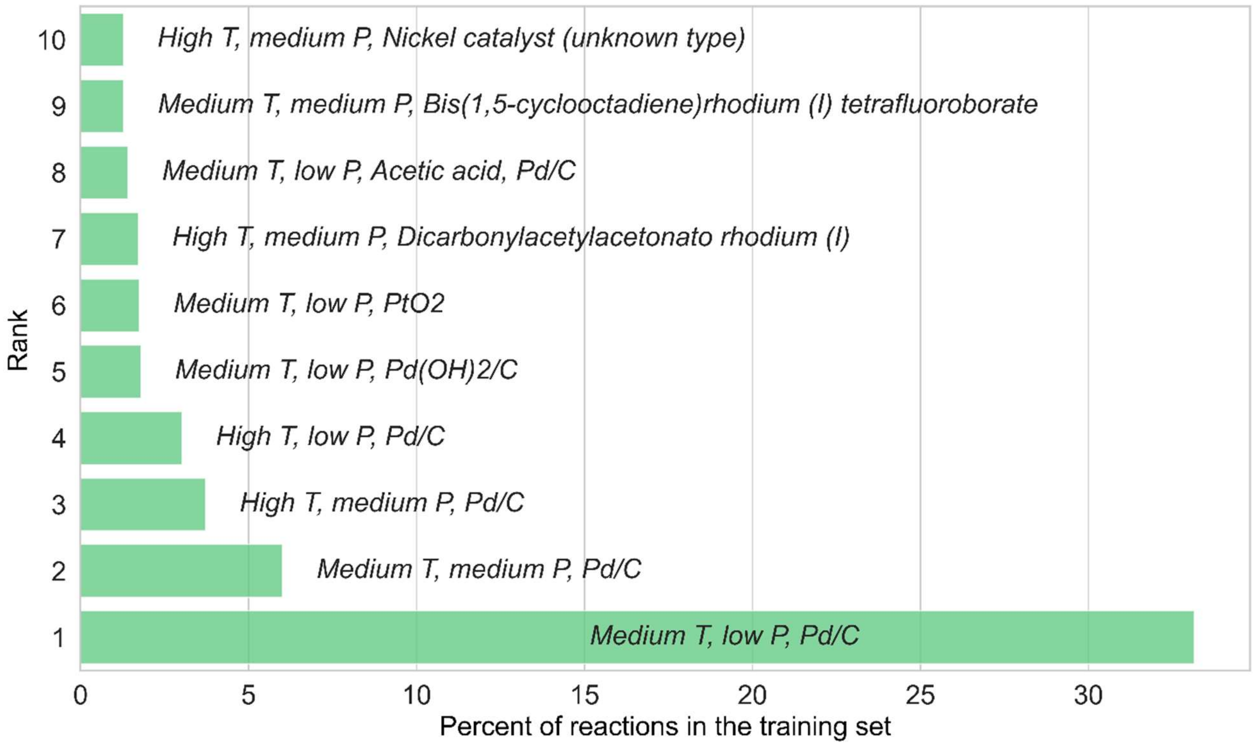

Figure S1. Top-10 most frequent conditions in the training set for dataset B. Conditions with unknown temperature or pressure were ignored (temperature: low - less 0 °C, medium - 0-40 °C, high - more 40 °C; pressure: very low – less 1 atm, low - 1-3.5 atm, medium – 3.5-100 atm, high - more 100 atm).

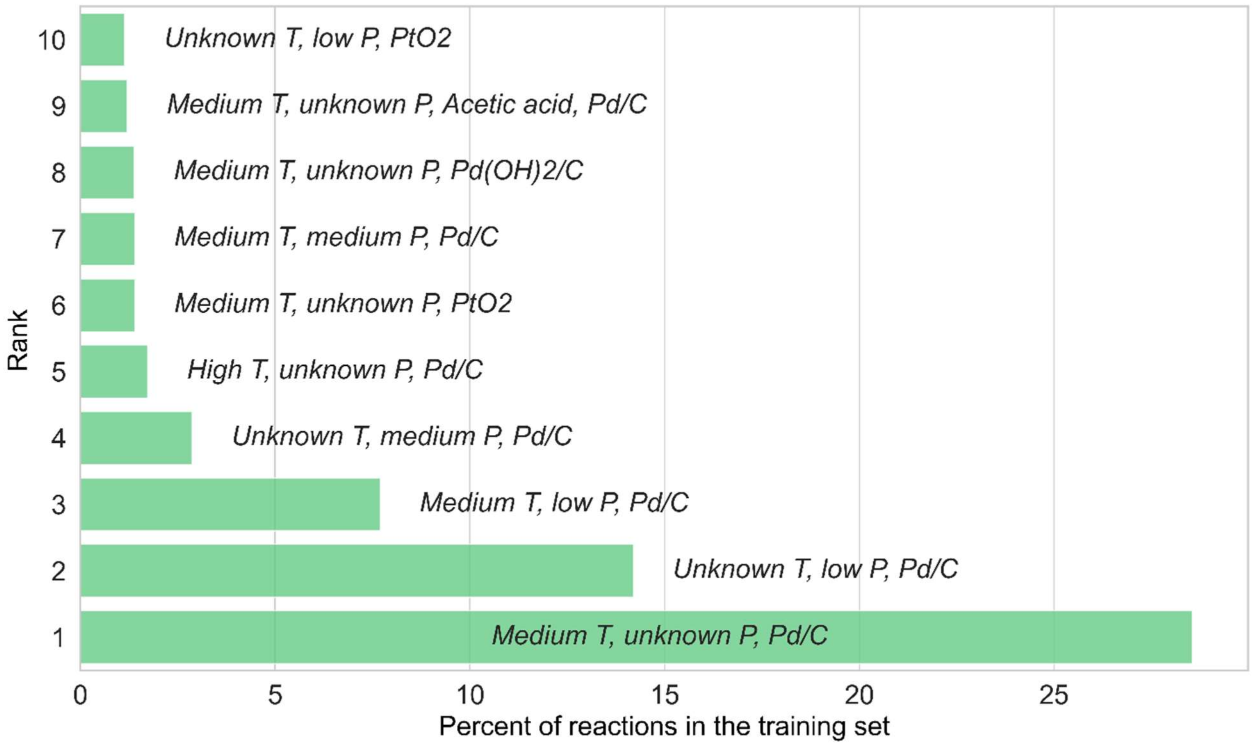

Figure S2. Top-10 most frequent conditions in the training set for dataset B. Conditions with unknown temperature or pressure were not ignored. (temperature: low - less 0 °C, medium - 0-40 °C, high - more 40 °C. pressure: very low – less 1 atm, low - 1-3.5 atm, medium – 3.5-100 atm, high - more 100 atm).

Table S1. Example of h-CVAE model predictions.

№	Reaxys© ID	Reaction	Ground-truth reaction condition	Predicted conditions		
				Top-1	Top-2	Top-3
1	4203709	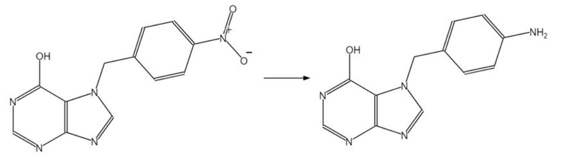	t=25,<br>p=3.2,<br>Acetic acid,<br>Palladium on activated carbon	10<=t<=40,<br>1<=p<=3.5, Acetic acid,<br>Palladium on activated carbon	10<=t<=40,<br>1<=p<=3.5,<br>Palladium on activated carbon	10<=t<=40,<br>3.5<p<100,<br>Acetic acid,<br>Palladium on activated carbon
2	36272873	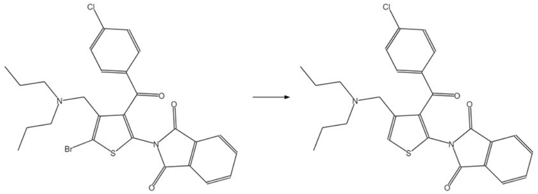	t=20,<br>p=4.1,<br>N,N-diethylethanamine,<br>Palladium on activated carbon	10<=t<=40,<br>3.5<p<100,<br>N,N-diethylethanamine,<br>N,N-dimethylformamide,<br>Palladium on activated carbon	10<=t<=40,<br>1<=p<=3.5,<br>N,N-diethylethanamine,<br>N,N-dimethylformamide,<br>Palladium on activated carbon	t>40,<br>3.5<p<100,<br>N,N-diethylethanamine,<br>N,N-dimethylformamide,<br>Palladium on activated carbon
3	46580796	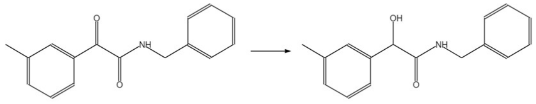	t=20,<br>p=40.0,<br>Chloro(1,5-cyclooctadiene)iridium(I) dimer	10<=t<=40,<br>3.5<p<100,<br>Chloro(1,5-cyclooctadiene)iridium(I) dimer	10<=t<=40,<br>1<=p<=3.5,<br>Chloro(1,5-cyclooctadiene)iridium(I) dimer	t>40,<br>3.5<p<100,<br>Chloro(1,5-cyclooctadiene)iridium(I) dimer
4	9051636	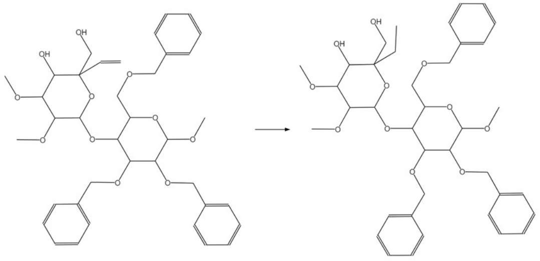	t=20,<br>p=1.4,<br>Platinum (IV) oxide	10<=t<=40,<br>1<=p<=3.5,<br>Platinum (IV) oxide	10<=t<=40,<br>1<=p<=3.5,<br>Palladium on activated carbon	t>40,<br>3.5<p<100,<br>Palladium on activated carbon
5	50123302	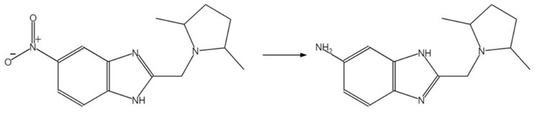	t=23,<br>p=1.0,<br>Palladium on activated carbon	10<=t<=40,<br>1<=p<=3.5,<br>Palladium on activated carbon	10<=t<=40,<br>3.5<p<100,<br>Palladium on activated carbon	10<=t<=40,<br>1<=p<=3.5,<br>Nickel catalyst

№	Reaxys© ID	Reaction	Ground-truth reaction condition	Predicted conditions		
				Top-1	Top-2	Top-3
6	2668158	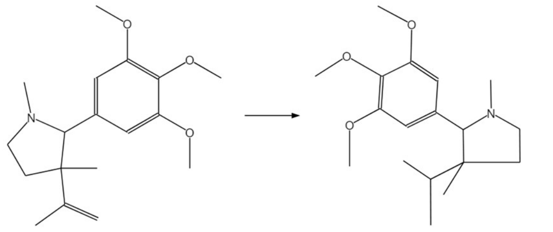	t=25, p=1.0, Palladium on activated carbon	10<=t<=40, 1<=p<=3.5, Palladium on activated carbon	10<=t<=40, 3.5<p<100, Palladium on activated carbon	t>40, 3.5<p<100, Palladium on activated carbon
7	2458983	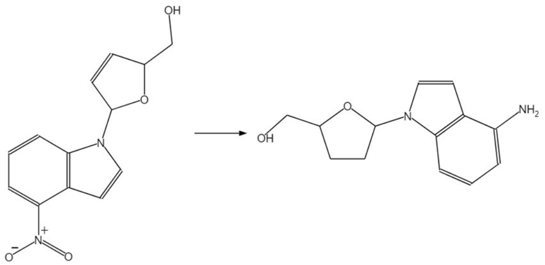	t=25, p=1.0, Palladium on activated carbon	10<=t<=40, 1<=p<=3.5, Palladium on activated carbon	10<=t<=40, 3.5<p<100, Palladium on activated carbon	10<=t<=40, 1<=p<=3.5, Nickel catalyst
8	23845785	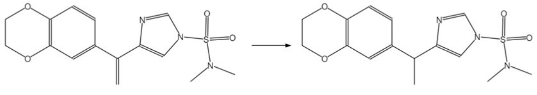	t=20, p=2.7, Palladium on activated carbon	10<=t<=40, 1<=p<=3.5, Palladium on activated carbon	10<=t<=40, 3.5<p<100, Palladium on activated carbon	10<=t<=40, 1<=p<=3.5, Platinum (IV) oxide
9	5028845	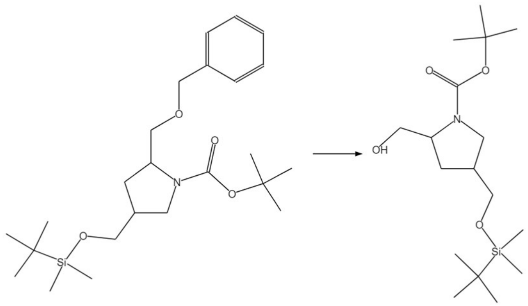	t=25, p=1.0, Palladium on activated carbon	10<=t<=40, 1<=p<=3.5, Palladium on activated carbon	10<=t<=40, 3.5<p<100, Palladium on activated carbon	10<=t<=40, 1<=p<=3.5, Nickel catalyst
10	27993032	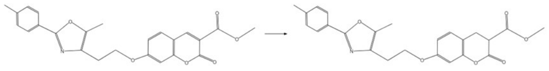	t=20, p=5.0, Palladium on activated carbon	10<=t<=40, 3.5<p<100, Palladium on activated carbon	10<=t<=40, 3.5<p<100, Platinum (IV) oxide	10<=t<=40, 1<=p<=3.5, Palladium on activated carbon

№	Reaxys© ID	Reaction	Ground-truth reaction condition	Predicted conditions		
				Top-1	Top-2	Top-3
11	23593473	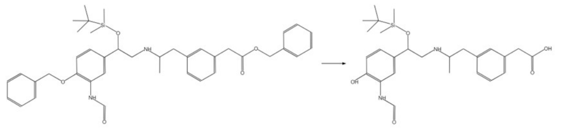	t=20,<br>p=4.1,<br>Palladium on activated carbon	10<=t<=40,<br>3.5<p<100,<br>Palladium on activated carbon	t>40,<br>3.5<p<100,<br>Palladium on activated carbon	10<=t<=40,<br>1<=p<=3.5,<br>Nickel catalyst
12	32370001	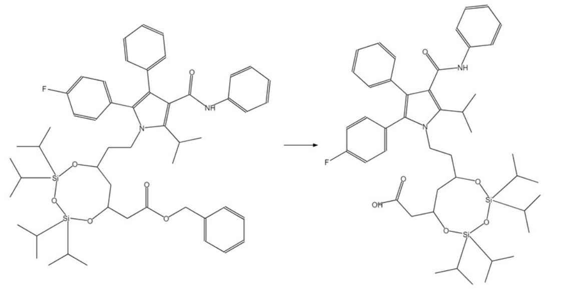	t=20,<br>p=3.9,<br>Palladium on activated carbon	10<=t<=40,<br>3.5<p<100,<br>Palladium on activated carbon	t>40,<br>3.5<p<100,<br>Nickel catalyst	t>40,<br>3.5<p<100,<br>Palladium on activated carbon
13	9345906	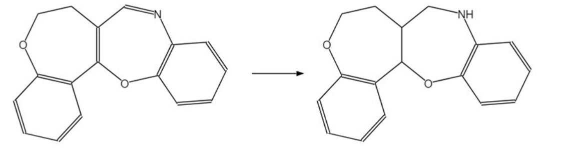	t=60,<br>p=7.5,<br>Palladium on activated carbon	t>40,<br>3.5<p<100,<br>Palladium on activated carbon	10<=t<=40,<br>1<=p<=3.5,<br>Palladium on activated carbon	10<=t<=40,<br>3.5<p<100,<br>Palladium on activated carbon
14	2755520	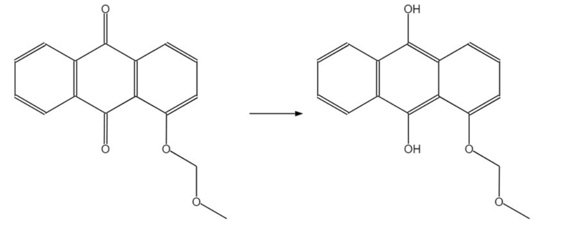	t=25,<br>p=1.0,<br>N,N-dimethylformamide,<br>Palladium on activated carbon	10<=t<=40,<br>1<=p<=3.5,<br>N,N-dimethylformamide,<br>Palladium on activated carbon	10<=t<=40,<br>1<=p<=3.5,<br>N,N-dimethylformamide,<br>Nickel catalyst	10<=t<=40,<br>1<=p<=3.5,<br>Palladium on activated carbon
15	37066674	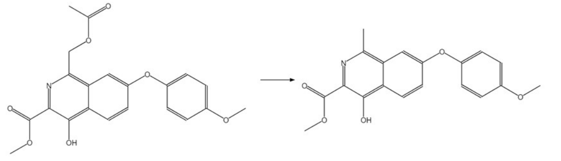	t=60,<br>p=4.1,<br>Palladium on activated carbon	t>40,<br>3.5<p<100,<br>Palladium on activated carbon	t>40,<br>1<=p<=3.5,<br>Palladium on activated carbon	10<=t<=40,<br>3.5<p<100,<br>Palladium on activated carbon

№	Reaxys© ID	Reaction	Ground-truth reaction condition	Predicted conditions		
				Top-1	Top-2	Top-3
16	11251069	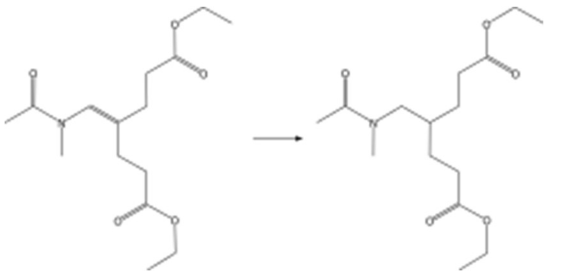	t=20, p=1, Platinum (IV) oxide	10<=t<=40, 1<=p<=3.5, Platinum (IV) oxide	10<=t<=40, 1<=p<=3.5, Palladium on activated carbon	10<=t<=40, 3.5<p<100, Palladium on activated carbon
17	28515631	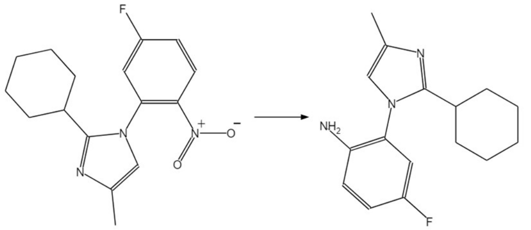	t=42.5, p=9.9, Nickel catalyst	t>40, 3.5<p<100, Nickel catalyst	t>40, 3.5<p<100, Palladium on activated carbon	t>40, 3.5<p<100, Platinum (IV) oxide
18	3158971	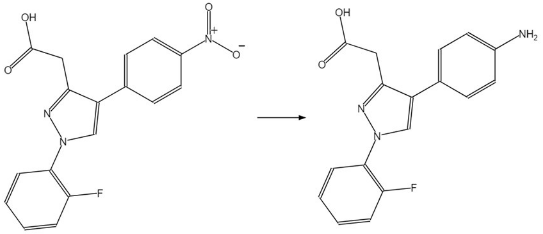	t=25, p=96.8, Nickel catalyst	10<=t<=40, 3.5<p<100, Nickel catalyst	10<=t<=40, 1<=p<=3.5, Palladium on activated carbon	10<=t<=40, 3.5<p<100, Palladium on activated carbon
19	3254648	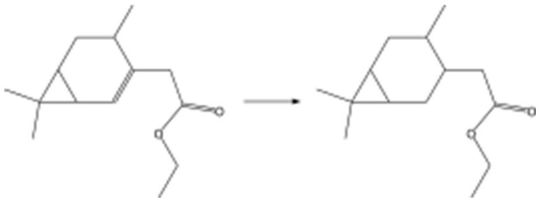	t=70, p=69.1, Nickel catalyst	t>40, 3.5<p<100, Nickel catalyst	t>40, 3.5<p<100, Palladium on activated carbon	t>40, 3.5<p<100, Bis(2-methylallyl)(1,5-cyclooctadiene)ruthenium(II)
20	4109945	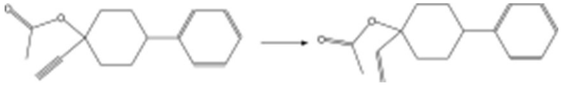	t=20, p=1.0, Lindlar catalyst	10<=t<=40, 1<=p<=3.5, Lindlar catalyst	10<=t<=40, 1<=p<=3.5, Dinickel boride	10<=t<=40, 1<=p<=3.5, Palladium on barium sulfate

№	Reaxys© ID	Reaction	Ground-truth reaction condition	Predicted conditions		
				Top-1	Top-2	Top-3
21	3703903	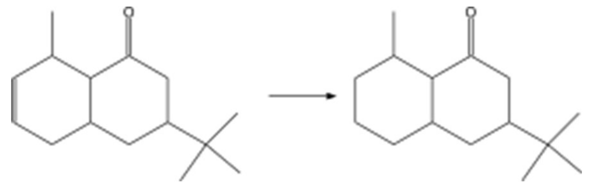	t=25, p=1, Platinum (IV) oxide	10<=t<=40, 1<=p<=3.5, Platinum (IV) oxide	10<=t<=40, 1<=p<=3.5, Palladium on activated carbon	10<=t<=40, 1<=p<=3.5, Nickel catalyst
22	39313437	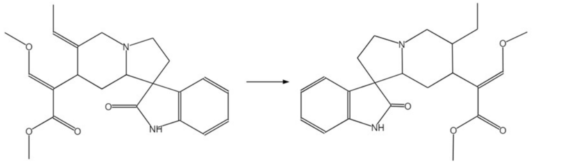	t=20, p=2.7, Platinum (IV) oxide	10<=t<=40, 1<=p<=3.5, Platinum (IV) oxide	10<=t<=40, 1<=p<=3.5, Nickel catalyst	10<=t<=40, 3.5<p<100, Nickel catalyst
23	4042630	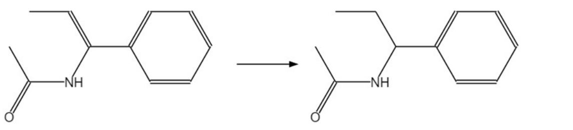	t=20, p=19.7, Bis(1,5-cyclooctadiene)rhodium(I) tetrafluoroborate	10<=t<=40, 3.5<p<100, Bis(1,5-cyclooctadiene)rhodium(I) tetrafluoroborate	10<=t<=40, 1<=p<=3.5, Bis(1,5-cyclooctadiene)rhodium(I) tetrafluoroborate	t>40, 3.5<p<100, Bis(1,5-cyclooctadiene)rhodium(I) tetrafluoroborate
24	33432732	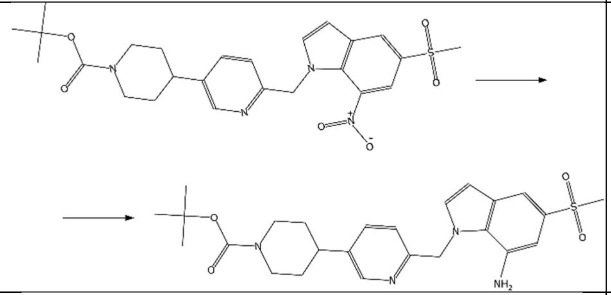	t=20, p=1, Platinum (IV) oxide	10<=t<=40, 1<=p<=3.5, Platinum (IV) oxide	10<=t<=40, 1<=p<=3.5, Nickel catalyst	10<=t<=40, 1<=p<=3.5, Palladium on activated carbon
25	46825012	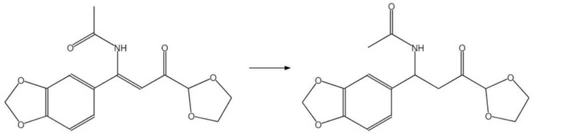	t=40, p=1.0, Bis(1,5-cyclooctadiene)rhodium(I) tetrafluoroborate	10<=t<=40, 1<=p<=3.5, Bis(1,5-cyclooctadiene)rhodium(I) tetrafluoroborate	10<=t<=40, 3.5<p<100, Bis(1,5-cyclooctadiene)rhodium(I) tetrafluoroborate	10<=t<=40, 1<=p<=3.5, Palladium on activated carbon

Table S2. Example of model predictions compared with recorded context test set where the h-CVAE model correctly identified the combination of RC, with the exception of one condition.

№	Reaxys© ID	Reaction	Ground-truth reaction condition	Predicted conditions		
				**Top-1**	**Top-2**	**Top-3**
**The same catalyst in top-1**						
3	3058357	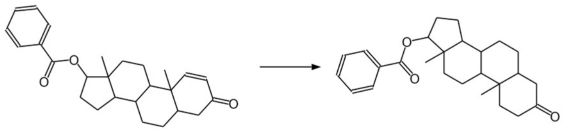	t=20, p=1, Lindlar catalyst	10<=t<=40, 1<=p<=3.5, Palladium on barium sulfate	10<=t<=40, 3.5<p<100, Lindlar catalyst	10<=t<=40, 1<=p<=3.5, Lindlar catalyst
4	2226094	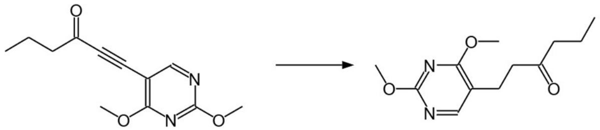	t=20, p=1, Palladium on activated carbon	10<=t<=40, 1<=p<=3.5, Lindlar catalyst	10<=t<=40, 1<=p<=3.5, Quinoline, Lindlar catalyst	10<=t<=40, 1<=p<=3.5, Palladium on barium sulfate
5	2568436	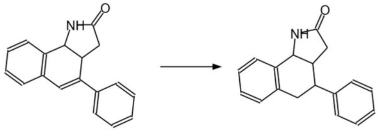	t=25, p=3, Palladium on activated carbon	10<=t<=40, 1<=p<=3.5, Palladium on calcium carbonate	10<=t<=40, 1<=p<=3.5, Palladium on activated carbon	10<=t<=40, 3.5<p<100, Palladium on calcium carbonate
7	2775929	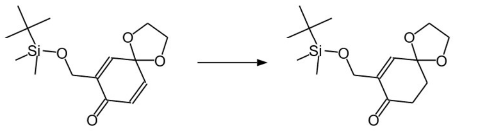	t=23, p=1, Rhodium on activated carbon	10<=t<=40, 1<=p<=3.5, Bis(norbornadiene)rhodium(i) tetrafluoroborate	10<=t<=40, 3.5<p<100, Bis(norbornadiene)rhodium(i) tetrafluoroborate	10<=t<=40, 1<=p<=3.5, Di-μ-chloro-bis(1,5-cyclooctadiene)dirhodium
8	2840626	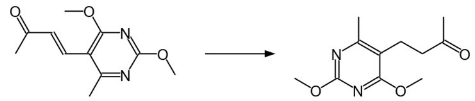	t=20, p=1, Palladium on activated carbon	10<=t<=40, 1<=p<=3.5, Palladium (ii) oxide on barium sulfate	10<=t<=40, 3.5<p<100, Palladium (ii) oxide on barium sulfate	t>40, 1<=p<=3.5, Palladium (ii) oxide on barium sulfate
9	3070334	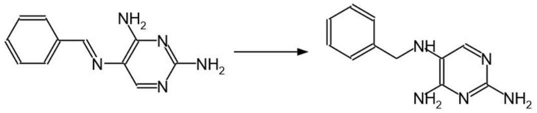	t=20, p=1, Palladium on activated carbon	10<=t<=40, 1<=p<=3.5, Palladium on calcium carbonate	10<=t<=40, 1<=p<=3.5, Palladium on activated carbon	10<=t<=40, 1 <=p<=3.5, Lindlar catalyst

№	Reaxys© ID	Reaction	Ground-truth reaction condition	Predicted conditions		
				Top-1	Top-2	Top-3
**Another base in top-1**						
10	37270750	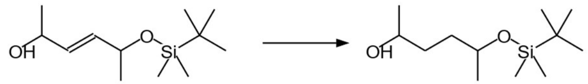	t=20,<br>p=3.5,<br>Potassium hydroxide,<br>Palladium on activated carbon	10<=t<=40,<br>1<=p<=3.5,<br>N,n-diethylethanamine,<br>Palladium on activated carbon	10<=t<=40,<br>1<=p<=3.5,<br>Palladium on activated carbon	10<=t<=40,<br>1<=p<=3.5,<br>N,n-diethylethanamine,<br>Palladium (ii) hydroxide on activated carbon
**Another pressure in top-1**						
11	48199695	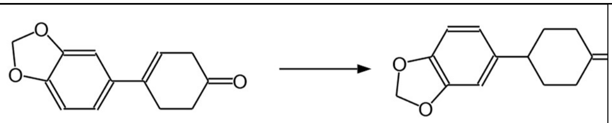	t=20,<br>p=4,<br>Palladium on activated carbon	10<=t<=40,<br>1<=p<=3.5,<br>Palladium on activated carbon	10<=t<=40,<br>3.5<p<100,<br>Palladium on activated carbon	10<=t<=40, 1<br><=p<=3.5,<br>Nickel catalyst
12	2481893	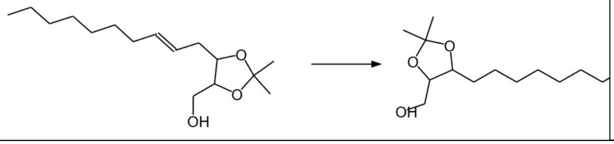	t=24,<br>p=1,<br>Palladium on activated carbon	10<=t<=40,<br>3.5<p<100,<br>Palladium on activated carbon	t>40,<br>3.5<p<100,<br>Palladium on activated carbon	10<=t<=40,<br>3.5<p<100,<br>Nickel catalyst
**Another temperature in top-1**						
13	30807383	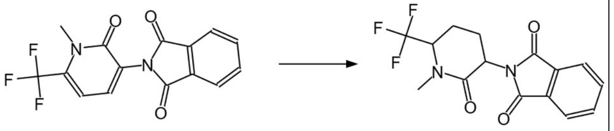	t=20,<br>p=30,<br>Palladium on activated carbon	t>40,<br>3.5<p<100,<br>Palladium on activated carbon	t>40,<br>3.5<p<100,<br>Ruthenium on activated carbon	t>40,<br>3.5<p<100,<br>Platinum on activated carbon
14	33253227	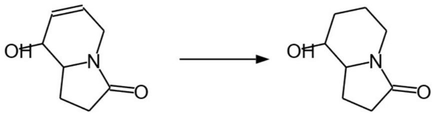	t=20,<br>p=5,<br>Palladium on activated carbon	t>40,<br>3.5<p<100,<br>Palladium on activated carbon	t>40,<br>1<=p<=3.5,<br>Palladium on activated carbon	t>40,<br>3.5<p<100,<br>Nickel catalyst
15	3258836	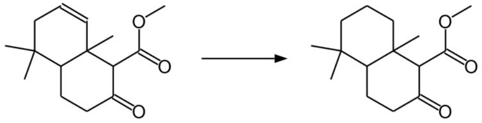	t=20,<br>p=1,<br>Palladium on activated carbon	t>40,<br>1<=p<=3.5,<br>Palladium on activated carbon	t>40,<br>1<=p<=3.5,<br>Nickel catalyst	10<=t<=40,<br>1<=p<=3.5,<br>Palladium on activated carbon

№	Reaxys© ID	Reaction	Ground-truth reaction condition	Predicted conditions		
				Top-1	Top-2	Top-3
			**Predicted acid in top-1**			
17	9159807	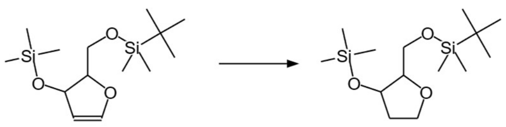	t=20,<br>p=1,<br>Palladium on activated carbon	10<=t<=40,<br>1<=p<=3.5,<br>Perchloric acid,<br>Palladium on activated carbon	10<=t<=40,<br>3.5<p<100,<br>Perchloric acid,<br>Palladium on activated carbon	10<=t<=40,<br>1<=p<=3.5,<br>Perchloric acid,<br>Palladium dihydroxide
18	9998378	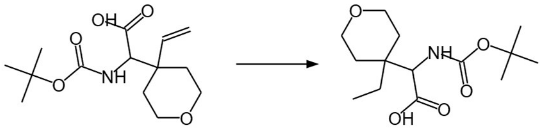	t=20,<br>p=1,<br>Palladium on activated carbon	10<=t<=40,<br>1<=p<=3.5,<br>Phosphoric acid,<br>Palladium on activated carbon	t>40,<br>3.5<p<100,<br>Phosphoric acid,<br>Palladium on activated carbon	10<=t<=40,<br>3.5<p<100,<br>Phosphoric acid,<br>Palladium on activated carbon
19	25851278	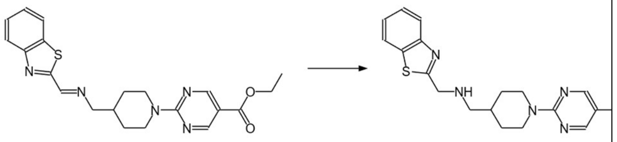	t=20,<br>p=3,<br>Palladium on activated carbon	10<=t<=40,<br>1<=p<=3.5,<br>Acetic acid,<br>Palladium on activated carbon	10<=t<=40,<br>1<=p<=3.5,<br>Palladium on activated carbon	10<=t<=40,<br>3.5<p<100,<br>Acetic acid,<br>Palladium on activated carbon
21	38301711	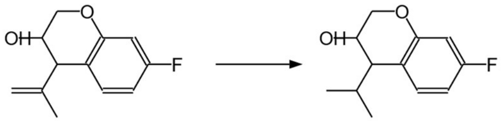	t=20,<br>p=3.5,<br>Palladium on activated carbon	10<=t<=40,<br>1<=p<=3.5,<br>Acetic acid,<br>Palladium on activated carbon	10<=t<=40,<br>3.5<p<100,<br>Acetic acid,<br>Palladium on activated carbon	t>40,<br>3.5<p<100,<br>Acetic acid,<br>Palladium on activated carbon
			**Predicted base in top-1**			
22	3561811	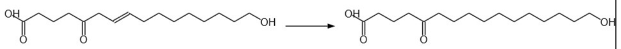	t=20,<br>p=2,<br>Palladium on activated carbon	10<=t<=40,<br>1<=p<=3.5,<br>N,n-diethylethanamine,<br>Palladium on activated carbon	10<=t<=40,<br>1<=p<=3.5,<br>N,n-diethylethanamine,<br>Platinum (iv) oxide	10<=t<=40,<br>1<=p<=3.5,<br>Palladium on activated carbon
23	35075928	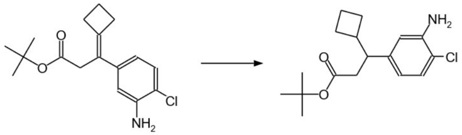	t=20,<br>p=1,<br>Palladium on activated carbon	10<=t<=40,<br>1<=p<=3.5,<br>Ammonia,<br>Palladium on activated carbon	10<=t<=40,<br>3.5<p<100,<br>Ammonia,<br>Palladium on activated carbon	10<=t<=40,<br>1<=p<=3.5,<br>Ammonia,<br>Nickel catalyst
24	2134983	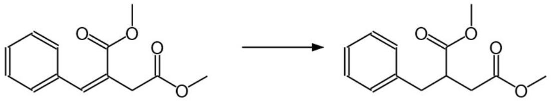	t=35,<br>p=2,<br>Palladium on activated carbon	10<=t<=40,<br>1<=p<=3.5,<br>Potassium hydroxide,<br>Palladium on activated carbon	10<=t<=40,<br>1<=p<=3.5,<br>Potassium hydroxide,<br>Nickel catalyst	10<=t<=40,<br>1<=p<=3.5,<br>Potassium hydroxide,<br>Platinum (iv) oxide

№	Reaxys© ID	Reaction	Ground-truth reaction condition	Predicted conditions		
				Top-1	Top-2	Top-3
24	33581924	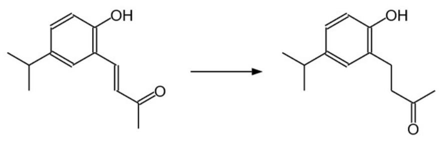	t=20, p=1, Palladium on activated carbon	10<=t<=40, 1<=p<=3.5, Hydrazine, Palladium on activated carbon	t>40, 3.5<p<100, Hydrazine, Palladium on activated carbon	t>40, 1<=p<=3.5, Hydrazine, Palladium on activated carbon
**Predicted rare catalyst in top-1**						
26	4566820	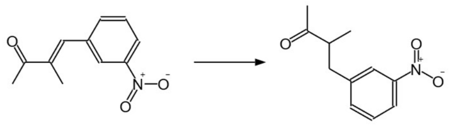	t=35, p=2, ((4s)-2-(2-(diphenylphosphino)phenyl)-4-tert-butyl-4,5-dihydrooxazole)-(η4-1,5-cyclooctadiene)iridium(i) tetrakis(3,5-bis(trifluoromethyl)phenyl)borate	10<=t<=40, 1<=p<=3.5, Palladium on activated carbon	t>40, 1<=p<=3.5, Palladium on activated carbon	t>40, 1<=p<=3.5, Palladium dihydroxide
27	4582877	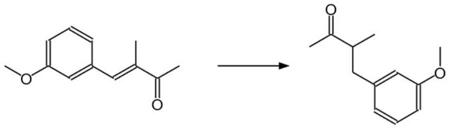	t=35, p=2, ((4s)-2-(2-(diphenylphosphino)phenyl)-4-tert-butyl-4,5-dihydrooxazole)-(η4-1,5-cyclooctadiene)iridium(i) tetrakis(3,5-bis(trifluoromethyl)phenyl)borate	10<=t<=40, 1<=p<=3.5, Palladium on activated carbon	t>40, 1<=p<=3.5, Palladium on activated carbon	10<=t<=40, 1<=p<=3.5, Nickel catalyst
28	28389193	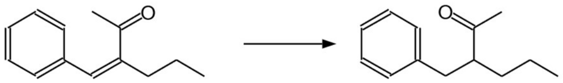	t=20, p=2, ((4s)-2-(2-(diphenylphosphino)phenyl)-4-tert-butyl-4,5-dihydrooxazole)-(η4-1,5-cyclooctadiene)iridium(i) tetrakis(3,5-bis(trifluoromethyl)phenyl)borate	10<=t<=40, 1<=p<=3.5, Palladium on activated carbon	t>40, 1<=p<=3.5, Palladium on activated carbon	t>40, 3.5<p<100, Palladium on activated carbon

№	Reaxys© ID	Reaction	Ground-truth reaction condition	Predicted conditions		
				Top-1	Top-2	Top-3
29	28389190	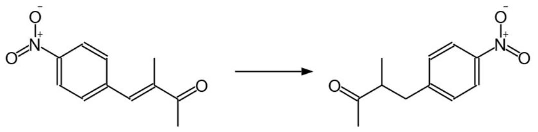	t=20, p=2, ((4s)-2-(2-(diphenylphosphino)phenyl)-4-tert-butyl-4,5-dihydrooxazole)-(η4-1,5-cyclooctadiene)iridium(i) tetrakis(3,5-bis(trifluoromethyl)phenyl)borate	10<=t<=40, 1<=p<=3.5 Palladium on activated carbon	10<=t<=40, 3.5<p<100, Palladium on activated carbon	10<=t<=40, 1<=p<=3.5, Palladium (ii) hydroxide on activated carbon
30	48990433	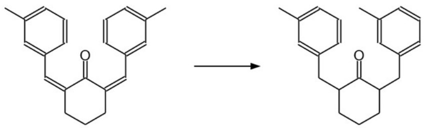	t=20, p=30, ((4s)-2-(2-(diphenylphosphino)phenyl)-4-tert-butyl-4,5-dihydrooxazole)-(η4-1,5-cyclooctadiene)iridium(i) tetrakis(3,5-bis(trifluoromethyl)phenyl)borate	10<=t<=40, 3.5<p<100, Palladium on activated carbon	10<=t<=40, 1<=p<=3.5, Acetic acid, Platinum (iv) oxide	10<=t<=40, 1<=p<=3.5, Palladium on activated carbon

Table S3. List of the most common catalysts in the training set of dataset S.

1. Platinum oxide(IV)
2. Platinum on carbon
3. Platinum on aluminium oxide
4. Platinum catalyst (unknown type)
5. Palladium on carbon
6. Palladium hydroxide(II)
7. Palladium hydroxide(II) on carbon
8. Palladium catalyst (unknown type)
9. Palladium on barium sulfate
10. Palladium(II) acetate
11. Palladium(II) 2,2,2-trifluoroacetate
12. Palladium(II) chloride
13. Ruthenium on carbon
14. (Benzene)ruthenium dichloride dimer
15. Dicarbonyl(acetylacetonato)rhodium(I)
16. [2'-(diphenylphosphanyl)-[1,1'-binaphthalene]-2-yl]diphenylphosphane cycloocta-1,5-diene tetrafluoroboranuide rhodium complex
17. Chloro(1,5-cyclooctadiene)rhodium (I) dimer
18. Rhodium on carbon
19. Chloridotris(triphenylphosphine)rhodium(I)
20. Rhodium on aluminium oxide
21. Bis(1,5-cyclooctadiene)rhodium(I) tetrafluoroborate
22. Bis(1,5-cyclooctadiene)rhodium(I) trifluoromethanesulfonate
23. Dirhodium tetraacetate
24. Bis(norbornadiene)rhodium(I) tetrafluoroborate
25. Tris(triphenylphosphine)rhodium carbonyl hydride
26. Rhodium catalyst (unknown type)
27. Nickel catalyst (unknown type)
28. Raney nickel catalyst
29. Bis(1,5-cyclooctadiene)diiridium(I) dichloride
30. Dicobalt octacarbonyl
31. Lindlar catalyst

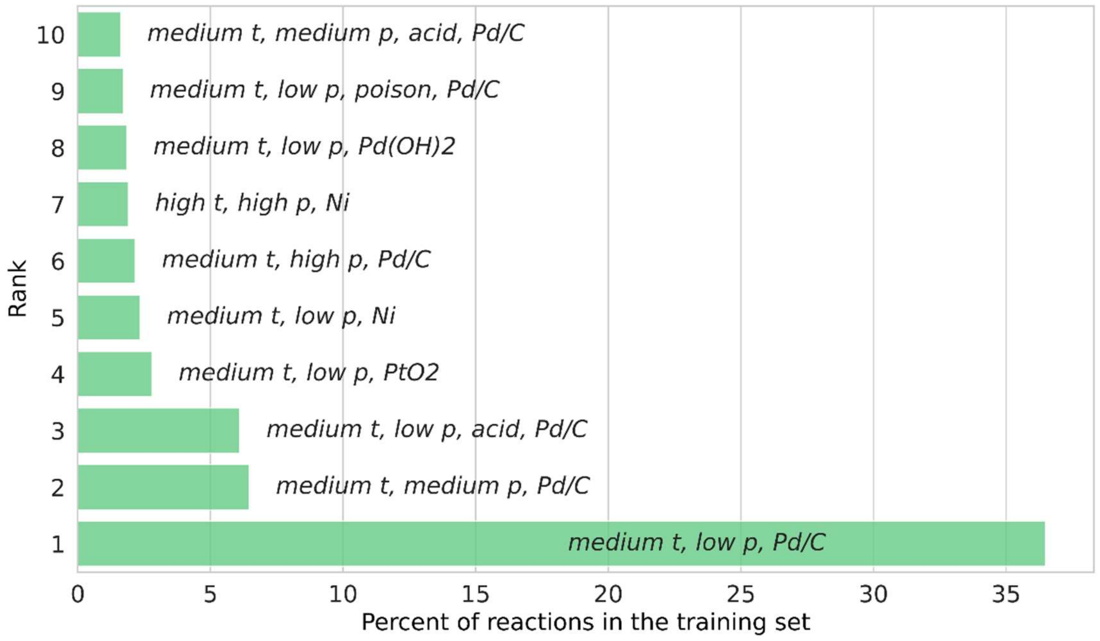

Figure S3. TOP-10 most frequent conditions in the training set for dataset S (temperature: low - less 0 °C, medium - 0-50 °C, high - more 50 °C; pressure: low - 0-3 atm, medium - 3-10 atm, high - more 10 atm).

Table S4. List of the reagents in the training set of dataset B.

1. Ethaneperoxoic acid
2. Acetic acid
3. (7,7-dimethyl-2-oxo-1-bicyclo[2.2.1]heptanyl)methanesulfonic acid
4. Propanoic acid
5. Methanesulfonic acid
6. 2,2,2-trifluoroacetic acid
7. Oxalic acid
8. 2,2,2-triphenylacetic acid
9. 2-[2-[bis(carboxymethyl)amino]ethyl-(carboxymethyl)amino]acetic acid
10. Benzoic acid
11. 2,3-dihydroxybutanedioic acid
12. Formic acid
13. Trifluoromethanesulfonic acid
14. Boric acid
15. Jones reagent
16. Sulfuric acid
17. Hydrogen sulfide
18. Hypophosphorous acid
19. Phosphoric acid
20. Tetrafluoroboric acid
21. Hydrogen bromide
22. Perchloric acid
23. Chlorane
24. Hydrogen fluoride
25. Hydrogen iodide
26. Nitric acid
27. N,n'-dicyclohexylmethanediimine
28. 1-(12,14-dioxa-13-phosphapentacyclo[13.8.0.02,11.03,8.018,23]tricosa-1(15),2(11),3,5,7,9,16,18,20,22-decaen-13-yl)piperidine
29. Pyrrolidine
30. Piperidine
31. 1,4-diazabicyclo[2.2.2]octane
32. 2,3,4,6,7,8,9,10-octahydropyrimido[1,2-a]azepine
33. N-propan-2-ylpropan-2-amine
34. 1-hydroxy-2,2,6,6-tetramethylpiperidine
35. Butan-1-amine
36. N,n-dibutylbutan-1-amine
37. N-ethyl-n-propan-2-ylpropan-2-amine
38. N,n-diethylethanamine
39. N-ethylethanamine
40. Methanamine
41. N,n-dimethylmethanamine
42. 1,1,3,3-tetramethylguanidine
43. 1,1,3,3-tetramethylthiourea
44. N,n,n',n'-tetramethylethane-1,2-diamine
45. N-[bis(dimethylamino)phosphoryl]-n-methylmethanamine
46. N,n-dimethylpyridin-4-amine
47. 4-methylmorpholine
48. N-methylmethanamine
49. 2,4,6-trimethylpyridine
50. 2,6-dimethylpyridine
51. [4-(5-diphenylphosphanyl-2,2-difluoro-1,3-benzodioxol-4-yl)-2,2-difluoro-1,3-benzodioxol-5-yl]-diphenylphosphane

52. Thiourea
53. Ethane-1,2-diamine
54. 2-aminoethanol
55. Phenylmethanamine
56. [1-[2-(12,14-dioxa-13-phosphapentacyclo[13.8.0.02,11.03,8.018,23]tricosa-1(15),2(11),3,5,7,9,16,18,20,22-decaen-13-yloxy)naphthalen-1-yl]naphthalen-2-yl]-diphenylphosphane
57. 1-[3-(2-oxopyrrolidin-1-yl)cyclobutyl]pyrrolidin-2-one
58. (5-ethenyl-1-azabicyclo[2.2.2]octan-2-yl)-quinolin-4-ylmethanol
59. 2-[bis(2-hydroxyethyl)amino]ethanol
60. Potassium;2-methylpropan-2-olate
61. Potassium;propan-2-olate
62. Sodium;2-methylpropan-2-olate
63. Sodium;ethanolate
64. Sodium;methanolate
65. Sodium;periodate
66. 2-pyridin-2-ylpyridine
67. Phenanthridine
68. Pyridine
69. Barium(2+) dihydroxide
70. Calcium dihydroxide
71. Potassium hydroxide
72. Lithium(1+) hydroxide
73. Magnesium(2+) dihydroxide
74. Hydrazine
75. Hydroxylamine
76. Ammonia
77. Ammonium hydroxide
78. Sodium hydroxide
79. 1-bromopyrrolidine-2,5-dione
80. Cyclohexa-1,4-diene
81. Cyclohexene
82. Morpholine
83. Ethene
84. Acetonitrile
85. 2-[2-[2-[5,8-dioxo-1,3-bis[2-(1-phenylethylcarbamoyl)phenyl]-1,3,6,7-tetrahydro-[1,2,4]diazaphospholo[1,2-a]pyridazin-2-yl]phenyl]-5,8-dioxo-3-[2-(1-phenylethylcarbamoyl)phenyl]-1,3,6,7-tetrahydro-[1,2,4]diazaphospholo[1,2-a]pyridazin-1-yl]-n-(1-phenylethyl)benzamide
86. Trimethylsilyl n-trimethylsilylethanimidate
87. Bis(2,4-pentanedionato)cobalt(II)
88. Tris(acetylacetonato)cobalt
89. Prop-1-ene
90. 2-methylbut-2-ene
91. Hex-1-ene
92. Tributylstannyl
93. Diethyl 2,6-dimethyl-1,4-dihydropyridine-3,5-dicarboxylate
94. N,n-dimethylacetamide
95. N,n-dimethylformamide
96. 1-methylpyrrolidin-2-one
97. Dicyclohexyl-[1-(2-methoxyphenyl)imidazol-2-yl]phosphane
98. 1,3,5-trichloro-1,3,5-triazinane-2,4,6-trione
99. 1-iodopyrrolidine-2,5-dione
100. Benzonitrile
101. 2-[2-(2-aminoethylsulfanyl)ethylsulfanyl]ethanol
102. Aniline
103. 6-diphenylphosphanyl-1h-pyridin-2-one

104. Triethylammonium formate
105. 1-hydroxybenzotriazole
106. (r)-n-diphenylphosphino-n-methyl-[(s)-2-(diphenylphosphino)ferrocenyl]ethylamine
107. Trisodium;3-bis(3-sulfonatophenyl)phosphanylbenzenesulfonate
108. Tetrabutylazanium;hydroxide
109. Lead(2+);diacetate
110. Lead(4+) tetraacetate
111. Zhaophos
112. Quinoline
113. Thiophene
114. Carbon dioxide
115. Carbon monoxide
116. Cadmium
117. Dicobalt octacarbonyl
118. Cobalt catalyst
119. Chromium(iii) oxide
120. Chromium dichloride
121. Chromium(vi) oxide
122. Chromium catalyst
123. Mercury
124. Molybdenum hexacarbonyl
125. Molybdenum (vi) oxide
126. Molybdenum
127. Nitrogen
128. Lead
129. Dichlorostannane
130. Tin
131. Sulfur
132. Rh(1+)(1,5-cyclooctadiene)(η6-c6h5b(1-)ph3)
133. Bis(bicyclo[2.2.1]hepta-2,5-diene)dichlorodirhodium
134. (1,5-cyclooctadiene)(pyridine)(tricyclohexylphosphine)iridium(i) tetrakis[3,5-bis(trifluoromethyl)phenyl]borate
135. Bis(cyclooctadiene)iridium(i) tetrakis(3,5-bis(trifluoromethyl)phenyl)borate
136. Chloro(1,5-cyclooctadiene)iridium(i) dimer
137. Bis(1,5-cyclooctadiene)nickel
138. Di-μ-chloro-bis(1,5-cyclooctadiene)dirhodium
139. Chloro(1,5-hexadiene)rhodium(i) dimer
140. Dichloro bis(acetonitrile) palladium(ii)
141. Bis(2-methylallyl)(1,5-cyclooctadiene)ruthenium(ii)
142. ((4s)-2-(2-(diphenylphosphino)phenyl)-4-tert-butyl-4,5-dihydrooxazole)-(η4-1,5-cyclooctadiene)iridium(i) tetrakis(3,5-bis(trifluoromethyl)phenyl)borate
143. Nickel(ii) acetylacetonate
144. Palladium(ii) acetylacetonate
145. Dicarbonylacetylacetonato rhodium (i)
146. Rhodium(iii) acetylacetonate
147. Tris(acetylacetonato)ruthenium(iii)
148. Dirhodium tetraacetate
149. Rucl2[(s)-xyl-p-phos][(s)-daipen], rucl[(r)-xyl-p-phos][(r)-daipen]
150. Methoxy(cyclooctadiene)rhodium(i) dimer
151. Tris(dibenzylideneacetone)dipalladiumchloroform complex
152. Bistriphenylphosphinepalladium(ii) chloride
153. Bis(benzonitrile)palladium(ii) dichloride
154. Bis(η3-allyl-μ-chloropalladium(ii))
155. Tris(triphenylphosphine)rhodium(i) chloride
156. Bis(tricyclohexylphosphine)benzylidene ruthenium (iv) dichloride

157. Tris(triphenylphosphine)ruthenium(ii) dichloride
158. {bis[2-(diphenylphosphino)ethyl]amine}carbonylchlorohydridoruthenium(ii)
159. Dichloro-bis-[2-(diphenylphosphino)ethylamine]ruthenium
160. Bis(cyclopentadienyl)titanium(iv) dichloride
161. (bicyclo[2.2.1]hepta-2,5-diene)[1,4-bis(diphenylphosphino)butane]rhodium(i) tetrafluoroborate
162. Bis(norbornadiene)rhodium(i) tetrafluoroborate
163. Bis(1,5-cyclooctadiene)rhodium(i) tetrafluoroborate
164. [1,4-bis(diphenylphosphino)butane](1,5-cyclooctadiene)rhodium(i) tetrafluoroborate
165. Rh/duanphos complex
166. (-)-1,2-bis-((2r,5r)-2,5-diethylphospholano)benzene(cyclooctadiene) rhodium(i) tetrafluoroborate, (+)-1,2-bis[(2s,5s)-2,5-diethylphospholano]benzene(cyclooctadiene)rhodium(i) tetrafluoroborate
167. Bis(1,5-cyclooctadiene)rhodium(i) hexafluorophosphate
168. (1,5-cyclooctadiene)(pyridine)(tricyclohexylphosphine) iridium (i) hexafluorophosphate
169. (1,5-cyclooctadiene)[bis(methyldiphenylphosphine)]iridium(i) hexafluorophosphate
170. Rhodium(i)(norborna-2,5-diene)2 hexafluoroantimonate
171. Di[(η-1,2,5,6)-1,5-cyclooctadiene] rhodium hexafluoroantimonate
172. Tris(dibenzylideneacetone)dipalladium
173. [(ir(h){(s)-[(4,4-bi-2,2-difluoro-1,3-benzodioxole)-5,5-diyl]bis(diphenylphosphine)})2(μ-cl)3]cl
174. (1,1'-bis(diphenylphosphino)ferrocene)palladium(ii) dichloride
175. (triphenylphosphine)copper(i) hydride hexamer
176. Carbonylhydridetris(triphenylphosphine)rhodium(i)
177. Carbonylchlorohydridetris(triphenylphosphine) ruthenium(ii)
178. Pentamethylcycloopentadienyliridium(iii) chloride dimer
179. Nickel diacetate
180. Bis(cycloocta-1,5-diene)rhodium(i) trifluoromethanesulfonate
181. (-)-1,2-bis-((2r,5r)-2,5-diethylphospholano)benzene(cyclooctadiene) rhodium(i) trifluoromethanesulfonate, (+)-1,2-bis-[(2s,5s)-2,5-diethylphospholano]benzene (1,5-cycloctadiene)rhodium(i) trifluoromethanesulfonate
182. Tetrapropylammonium perruthennate
183. Rhodium chloride tri(triphenylphosphine-meta-trisulfonate)
184. Palladium (ii) acetate
185. Palladium (ii) trifluoroacetate
186. [rucl(benzene){(2,2,2',2'-tetramethyl[4,4'-bibenzo[d][1,3]dioxole]-5,5'-diyl)bis(diphenylphosphine)}]cl
187. Rubarf(p-cymene)(r,r)-msdpen
188. Ru(trifluoromethanesulfonate)(n-(p-toluenesulfonyl)-1,2-diphenylethylenediamine)(η6-cymene)
189. Dichloro(p-cymene)ruthenium(ii) dimer
190. Dichloro(benzene)ruthenium(ii) dimer
191. Diiodo(p-cymene)ruthenium(ii) dimer
192. 1-hydroxytetraphenylcyclopentadienyl(tetraphenyl-2,4-cyclopentadien-1-one)-μ-hydrotetracarbonyldiruthenium(ii)
193. Tetrakis(triphenylphosphine)palladium
194. Palladium, iron, sodium, silver, rhenium catalyst on activated carbon
195. Palladium, silver on alumina
196. Nickel, aluminum, iron catalyst
197. Nickel, titanium, aluminum catalyst
198. Nickel, aluminum catalyst
199. Palladium, gold on alumina
200. Palladium, gold on activated carbon
201. Palladium, gold catalyst
202. Platinum, gold catalyst
203. Nickel, cobalt, copper catalyst
204. Platinum, cobalt catalyst
205. Rhodium, cobalt catalyst
206. Nickel, copper, chromite catalyst
207. Nickel, copper catalyst
208. Palladium, copper on activated carbon

209. Platinum, copper on alumina
210. Platinum, copper on activated carbon
211. Rhodium, copper catalyst
212. Palladium, iron on activated carbon
213. Platinum, copper catalyst on activated carbon
214. Hexachloroplatinic acid
215. Platinum, iridium on titanium dioxide
216. Iridium on calcium carbonate
217. Iridium on activated carbon
218. Iridium on silica
219. Iridium on zirconium dioxide
220. Iridium catalyst
221. Lindlar catalyst
222. Molybdenum catalyst poisoned with sulfur
223. Molybdenum, nickel catalyst poisoned with sulfur
224. Molybdenum, nickel on alumina
225. Molybdenum, rhenium catalyst
226. Nickel (ii) nitrate
227. Nickel, cobalt catalyst
228. Nickel, chromium catalyst
229. Copper, nickel catalyst
230. Nickel, molybdenum sulfide catalyst
231. Nickel, molybdenum catalyst
232. Nickel, platinum on activated carbon
233. Nickel, rhenium catalyst
234. Nickel, ruthenium catalyst
235. Nickel, tungsten catalyst
236. Nickel on silica/alumina
237. Nickel on alumina
238. Nickel on aluminophosphate
239. Nickel on activated carbon
240. Nickel on silica
241. Nickel on titanium dioxide
242. Nickel on zinc oxide
243. Dinickel boride
244. Nickel (iii) oxide
245. Nickel (ii) orthophosphate
246. Nickel boride
247. Nickel dibromide
248. Nickel dichloride
249. Nickel (ii) fluoride
250. Nickel (ii) oxide
251. Nickel (ii) sulphate
252. Nickel (ii) sulfide
253. Nickel catalyst
254. Palladium (ii) nitrate
255. Palladium (ii) hydroxide on activated carbon
256. Palladium, copper catalyst
257. Palladium, iron catalyst
258. Palladium, gallium catalyst
259. Palladium, platinum on activated carbon
260. Palladium, platinum catalyst
261. Palladium, rhodium on activated carbon
262. Palladium, rhodium catalyst
263. Palladium, ruthenium catalyst

264. Palladium, vanadium catalyst
265. Palladium on aluminum carbonate
266. Palladium on silica/alumina
267. Palladium on alumina
268. Palladium on aluminum oxyhydroxide
269. Palladium on aluminum
270. Palladium on barium carbonate
271. Palladium on barium sulfate/activated carbon
272. Palladium on barium sulfate
273. Palladium on calcium carbonate
274. Palladium on calcium fluoride poisoned with lead
275. Palladium on calcium sulfate
276. Palladium on activated carbon
277. Palladium on iron (iii) oxide
278. Palladium on iron (ii, iii) oxide
279. Palladium on magnesium carbonate
280. Palladium on magnesium oxide
281. Palladium on nickel (ii) oxide
282. Palladium on silica
283. Palladium on strontium carbonate
284. Palladium on titanium dioxide
285. Palladium on zinc oxide
286. Palladium on zirconium dioxide
287. Palladium (ii) chloride on activated carbon
288. Palladium (ii) chloride
289. Palladium hydride on activated carbon
290. Palladium (ii) iodide
291. Palladium (ii) oxide on barium sulfate
292. Palladium (ii) oxide on calcium carbonate
293. Palladium (ii) oxide on activated carbon
294. Palladium (iv) oxide on activated carbon
295. Palladium (iv) oxide
296. Palladium (ii) oxide
297. Palladium (ii) sulfate
298. Palladium sulfide
299. Palladium catalyst
300. Platinum hydroxide on activated carbon
301. Platinum, rhenium catalyst
302. Platinum, rhodium on activated carbon
303. Platinum, rhodium
304. Platinum, tin on alumina
305. Platinum, vanadium on activated carbon
306. Platinum, wolfram on alumina
307. Platinum on alumina
308. Platinum on barium sulfate
309. Platinum on activated carbon
310. Platinum on magnesium oxide
311. Platinum on silica
312. Platinum on titanium dioxide
313. Platinum (ii) bromide
314. Platinum (ii) chloride
315. Platinum (iv) chloride
316. Platinum (ii) oxide on activated carbon
317. Platinum (iv) oxide on alumina
318. Platinum (iv) oxide on activated carbon

319. Platinum (iv) oxide on silica
320. Platinum (iv) oxide
321. Platinum (vi) oxide
322. Platinum (ii) sulfide on activated carbon
323. Platinum (iv) sulfide on activated carbon
324. Platinum catalyst
325. Raney cobalt doped with chromium
326. Raney cobalt
327. Raney copper
328. Raney nickel
329. Ruthenium, rhenium on activated carbon
330. Rhodium, zinc catalyst
331. Rhodium on alumina
332. Rhodium on aluminum
333. Rhodium on activated carbon
334. Rhodium on magnesium oxide
335. Rhodium on silica
336. Nishimura's catalyst
337. Rhodium (iii) oxide
338. Dodecacarbonyltetrarhodium
339. Hexarhodium hexadecacarbonyl
340. Rhodium (iii) chloride
341. Rhodium (iii) iodide
342. Rhodium catalyst
343. Ruthenium on alumina
344. Ruthenium on aluminum
345. Ruthenium on activated carbon
346. Ruthenium on magnesium oxide
347. Ruthenium on silica
348. Ruthenium on titanium dioxide
349. Dodecacarbonyltriruthenium
350. Ruthenium (iv) oxide
351. Ruthenium catalyst
352. Urushibara nickel
353. Cu-doped zirconium dioxide
354. Palladium acetate on alumina calcined
355. Chiral iridium complex of type PIr(N)(COD) + BArF-
356. Palladium dihydroxide
357. Palladium on aluminum hydroxide
358. Palladium on barium hydroxide

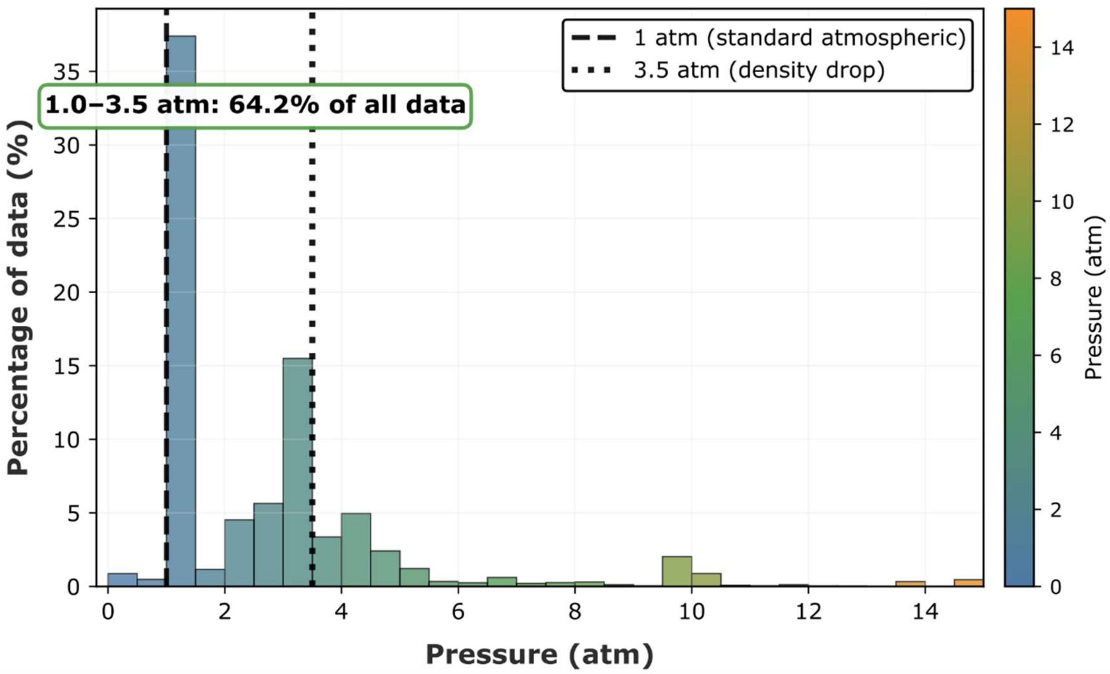

Figure S4: Distribution of pressure values in the training dataset B (detailed view: 0–15 atm range). The histogram (bin size: 0.5 atm) highlights the extreme data density in the 1–3.5 atm range, which contains 64.8% of all data. The sharp drop in frequency beyond 3.5 atm justifies its selection as a category threshold. Vertical dashed lines mark the 1 atm (standard atmospheric pressure) and 3.5 atm (empirical density drop) boundaries.

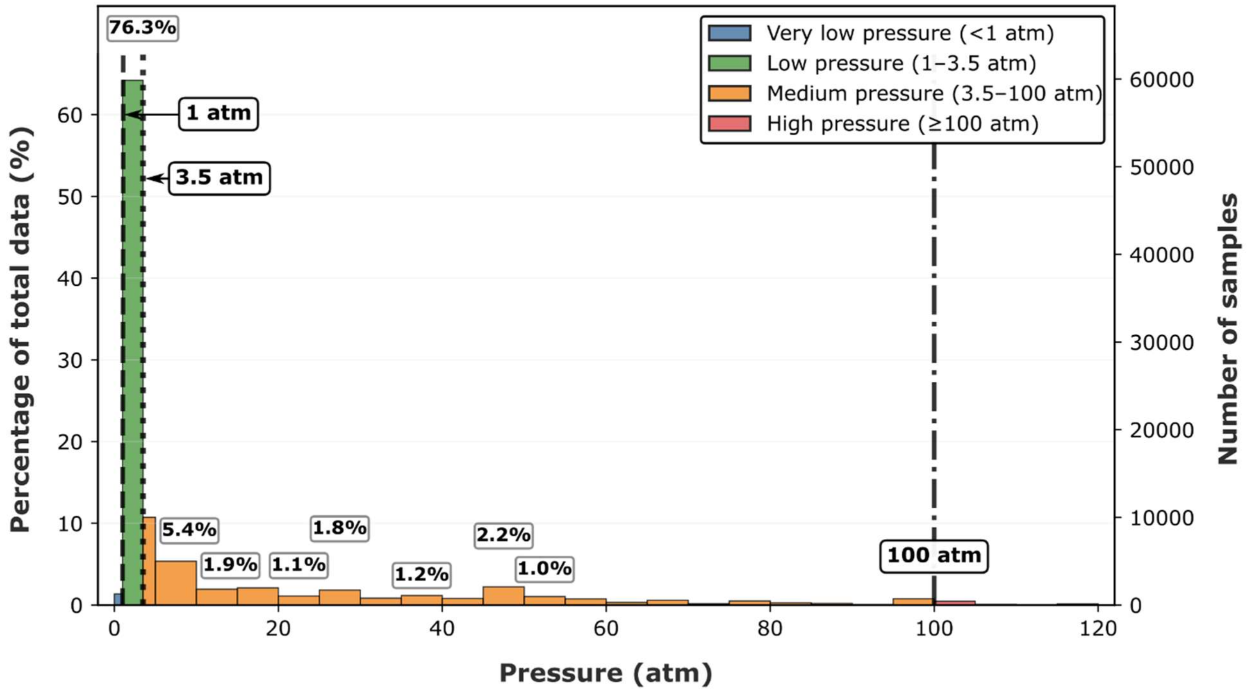

Figure S5. Complete distribution of pressure values in the training dataset B (overview: 0–120 atm range). The histogram uses variable-width bins to accurately represent the four defined pressure categories: very low (<1 atm, blue), low (1–3.5 atm, green), medium (3.5–100 atm, orange), and high (≥100 atm, red). Numbers above bars (shown for bins containing >1% of the data) indicate the percentage of total data in each 5-atm interval. The plot visually underscores the sharp decline in data density beyond 3.5 atm and the practical 100 atm limit for standard laboratory equipment.

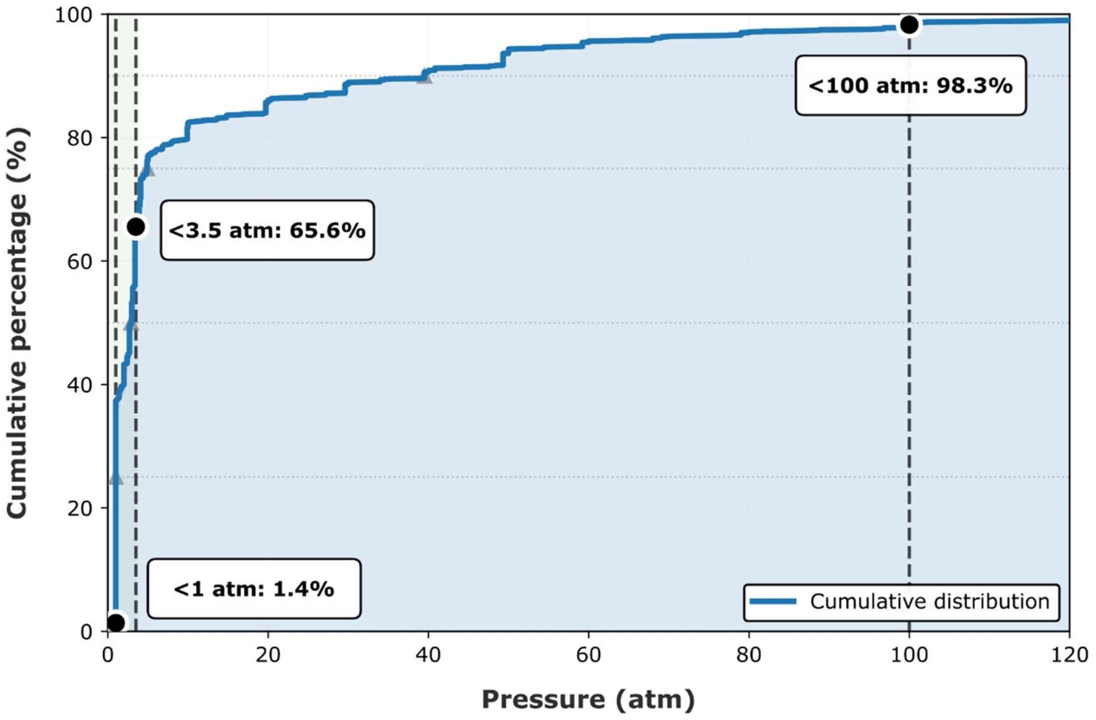

Figure S6. Cumulative distribution function (CDF) of pressure in the training dataset B. The curve shows the cumulative percentage of data with pressure below each value (i.e., P(pressure < x)), providing a statistical justification for the chosen category boundaries. Key thresholds are annotated: <1 atm (1.4% of data), <3.5 atm (65.6% of data), and <100 atm (98.3% of data). The 3.5 atm threshold approximates the point where ~75% of data have lower pressures, marking the transition from high-density to low-density data regions.
